# Origin of Enhanced Efficiency and Reduced Roll‐Off in Organic Light‐Emitting Diodes Based on Pt(II) Complexes

**DOI:** 10.1002/advs.77325

**Published:** 2026-08-24

**Authors:** Atul Shukla, Sarah K. M. McGregor, Julian A. Steele, Innes K. Gale, Kiyoshi Miyata, Monirul Hassan, Christian McDonald, Romain J. Lepage, Eduardo Solano, Pilankatta K. Ramya, Cherumuttathu H. Suresh, Takumi Ehara, Chihaya Adachi, Ken Onda, Shih‐Chun Lo, Ebinazar B. Namdas

**Affiliations:** ^1^ School of Mathematics and Physics The University of Queensland Brisbane Queensland Australia; ^2^ School of Chemistry and Molecular Biosciences The University of Queensland Brisbane Queensland Australia; ^3^ Australian Institute for Bioengineering and Nanotechnology The University of Queensland Brisbane Queensland Australia; ^4^ Department of Chemistry Kyushu University Fukuoka Japan; ^5^ NCD‐SWEET Beamline ALBA Synchrotron Light Source Barcelona Spain; ^6^ Chemical Sciences and Technology Division CSIR‐National Institute for Interdisciplinary Science and Technology Thiruvananthapuram Kerala India; ^7^ Srinivasa Ramanujan Institute for Basic Sciences Kottayam Kerala India; ^8^ Center For Organic Photonics and Electronics Research (OPERA) Kyushu University Fukuoka Japan

**Keywords:** EXAFS, metal‐metal‐to‐ligand charge‐transfer, OLEDs, square planar Pt(II) complex, ultrafast spectroscopy

## Abstract

Metal–metal‐to‐ligand charge‐transfer (MMLCT) complexes are emerging as next‐generation emitters that combine molecular precision with metallic coherence, achieving fast exciton decay and high photoluminescence quantum yields. Despite their promise, the lack of mechanistic insight linking molecular structure to exciton dynamics has hindered rational design. Here, we establish the microscopic origin of MMLCT emission in Pt(II) systems by integrating quantum‐chemical modelling, ultrafast spectroscopy, and synchrotron‐based x‐ray probes to deliver an end‐to‐end mechanistic picture of MMLCT‐state formation and decay. Direct measurements on **Pt(fppz)_2_** and its alkylated analogue **Pt(8ppz)_2_** quantify Pt–Pt spacings, revealing that few‐ångström variations govern the emergence and coherence of the MMLCT state. Coherently stacked **Pt(fppz)_2_** aggregates (Pt–Pt ≈3.3 Å) exhibit sub‐microsecond exciton decay with nearly complete exciton utilization, whereas **Pt(8ppz)_2_** (Pt–Pt >5 Å) yields long‐lived ligand‐centred emission. These contrasting exciton dynamics translate directly into different device performance, with **Pt(fppz)_2_**‐based organic light‐emitting diodes (OLEDs) achieving ∼29% external quantum efficiency and negligible roll‐off, enabling applications in visible‐light communication, bioimaging, and transparent displays. Our findings establish a generalizable structure–property relationship for MMLCT emitters, extending beyond Pt(II) to aggregated d^8^–d^8^ and coinage‐metal systems, unlocking new opportunities across optoelectronic and photonic technologies.

## Graphical abstract

Controlled Pt–Pt fine structure in square‐planar Pt(II) complexes governs metal–metal‐to‐ligand charge‐transfer (MMLCT) state formation, exciton dynamics, and organic light‐emitting diode performance. Short Pt–Pt contacts in **Pt(fppz)**_**2**_ enable coherent metal–metal coupling, sub‐microsecond emission and an external quantum efficiency of 29%, whereas increased Pt–Pt separation in **Pt(8ppz)**_**2**_ suppresses MMLCT formation, producing slower ligand‐centred/excimer‐like emission and poorer device performance.

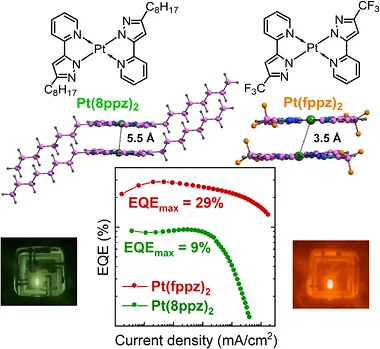

## 1 Introduction

Organic light‐emitting diodes (OLEDs) have undergone remarkable progress in recent years and are now widely used in display and lighting applications. Various organic light‐emitting semiconductors, including fluorescent [1, 2], phosphorescent [3, 4], and thermally activated delayed fluorescent (TADF) emitters [5, 6, 7, 8], have been successfully utilized in OLEDs. The key parameters for assessing OLED performance are brightness (measured in cd m^−2^) and internal quantum efficiency (IQE). For commercial TV displays, a minimum brightness of ≈500 cd m^−2^ is typically required. While fluorescent materials can achieve a maximum IQE of only 25% due to the non‐emissive triplet excited states, phosphorescent and TADF emitters can potentially reach 100% IQE by harnessing both singlet and triplet excitons via efficient intersystem crossing (ISC) and reverse ISC, respectively. Nonetheless, a significant challenge with the triplet‐based OLEDs is an adverse roll‐off effect, where device efficiency decreases dramatically as brightness levels increase.

Consequently, the desire to develop high‐brightness OLEDs with superior efficiency continues to mount, as this will enable emerging technologies such as semi‐transparent displays for augmented reality (AR) [9, 10], visible light communication [11], photodynamic cancer therapy [12], lasers [13, 14], imaging [15], and optogenetics [16]. A critical requirement of next‐generation emissive materials is to exhibit high IQE (≈100%) at elevated brightness levels (10 000 cd m^−2^), with both being fundamentally determined by the density and lifetimes of excitons within the device [17]. Because triplet excitons persist much longer than singlets, achieving high brightness with triplet‐based emitters necessitates elevated exciton densities. However, this regime enhances deleterious bimolecular interactions such as triplet‐triplet (TTA) and singlet‐triplet annihilation (STA), leading to parasitic efficiency roll‐off effects that are most pronounced in phosphorescent and TADF materials with long triplet lifetimes [18, 19, 20]. Addressing these challenges is essential for realizing the full potential of OLEDs in next‐generation optoelectronics.

Triplet emitters that combine high photoluminescence quantum yield (PLQY) with short excited‐state lifetimes represent a promising strategy to mitigate efficiency roll‐off in OLEDs. Within this context, recent advances have highlighted the promise of metal–metal‐to‐ligand charge‐transfer (MMLCT) complexes, which exhibit both shorter emission lifetimes and lower energy emission than traditional ligand‐to‐metal (LMCT) or metal‐to‐ligand charge‐transfer (MLCT) complexes [21, 22, 23]. Notably, MMLCT compounds exhibit substantial resistance to aggregation‐caused quenching, yielding high PLQYs and reduced excited‐state lifetimes even in neat films [24, 25]. OLEDs utilizing neat films of MMLCT Pt(II) emitters have demonstrated outstanding performance, particularly across the red to near‐infrared spectral regions [26, 27]. Crucially, efficient MMLCT transitions depend on short metal‐metal distances that foster overlap between p_z_ and dz2 orbitals of the adjacent Pt atoms, a relationship established by single‐crystal x‐ray crystallography [25, 28]. Nevertheless, high‐quality single crystals are challenging to obtain, and the thin evaporated films typically used in OLEDs generally display a semi‐crystalline phase. Consequently, single‐crystal analyses fail to capture the local Pt–Pt structure in thin films, a feature that profoundly influences excited‐state properties, exciton dynamics, and ultimately device performance. Due to their direct influence on OLED performance, controlling and understanding the intrinsic properties of Pt–Pt fine‐structure represents a scientifically challenging and technologically important *terra incognita*. In this work, we address this question by deliberately modifying the peripheral ligand structure to tune the local Pt–Pt separation while retaining the square‐planar Pt(II) coordination core.

**FIGURE 1:**
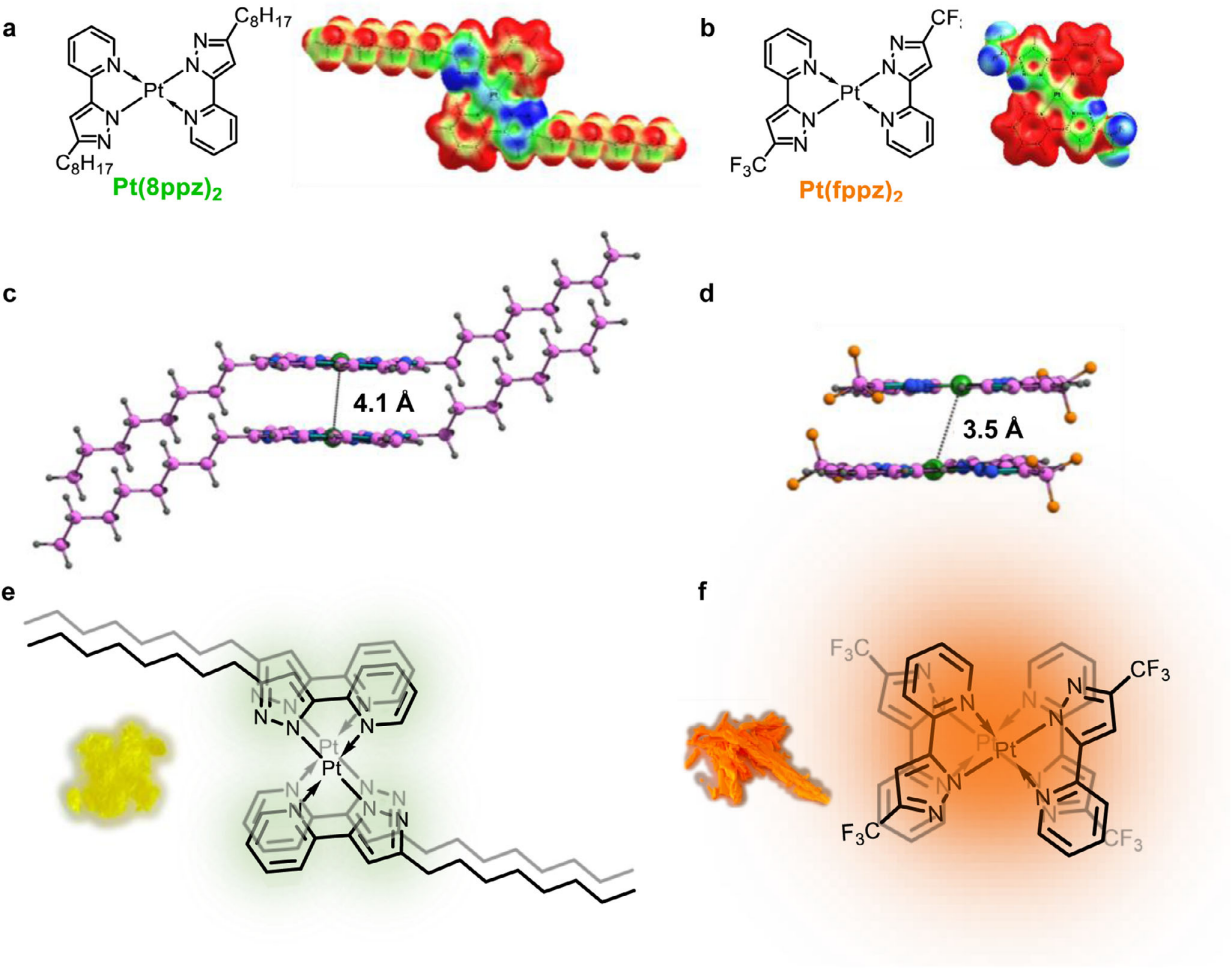
Molecular structures and electrostatic potential distributions of Pt(II) complexes. Molecular structures and molecular electrostatic potential (MESP) distributions mapped on the isodensity surface (0.01 a.u.) for the model Pt(II) complexes: (a) **Pt(8ppz)_2_** and (b) **Pt(fppz)_2_**. The color scale ranges from −20.0 to +20.0 kcal mol^−^^1^ (blue to red). Panels (c) and (d) present side views of the optimized dimer geometries of **Pt(8ppz)_2_** and **Pt(fppz)_2_**, respectively, obtained from QM calculations. Panels (e) and (f) show top‐view illustrations of the molecular stacking arrangements in **Pt(8ppz)_2_** and **Pt(fppz)_2_**, respectively, alongside images of their corresponding bulk solids.

Here, through controlled modulation of short‐range Pt–Pt interactions and local Pt fine structure, we establish their decisive role in governing the excited‐state dynamics, photophysics, and OLED performance of two structurally analogous square‐planar Pt(II) complexes. Using the well‐established **Pt(fppz)_2_** complex as a benchmark MMLCT emitter, we compare it to a tailored structural analogue, **Pt(8ppz)_2_**, which maintains the same core coordination geometry but replaces the electron‐withdrawing ─CF_3_ groups with n‐octyl chains. Quantum mechanical (QM) calculations predict that **Pt(8ppz)_2_** adopts a herringbone packing motif, driven by interdigitated alkyl chains, resulting in significantly increased Pt–Pt separations (Figure 1). Synchrotron‐based techniques, sensitive to both long‐range order (crystal packing) and short‐range Pt environments, confirm this physical distinction and that **Pt(fppz)_2_** possesses a short intermolecular Pt–Pt distance of 3.32 Å, enabling strong dz^2^ orbital overlap and efficient MMLCT emission. Close molecular packing further leads to near‐unity photoluminescence quantum yield (PLQY) and a short excited‐state lifetime of 370 ns in neat films. Ultrafast transient absorption (TA) measurements reveal coherent vibrational wavepacket motions of the Pt–Pt stretching mode in the MMLCT excited state of **Pt(fppz)_2_**. In contrast, **Pt(8ppz)_2_** exhibits Pt–Pt distances exceeding 5.5 Å, suppressing MMLCT and resulting in weak PLQY and prolonged excited‐state lifetimes, indicative of MLCT‐dominated emission. OLEDs based on **Pt(fppz)_2_** show exceptional performance, with peak EQEs of 29% and minimal roll‐off, maintaining an EQE of 23% at high brightness of 5000 cd m^−2^. These results establish a direct structure–property–device link, highlighting the critical role of molecular packing and Pt–Pt interactions in achieving high‐performance MMLCT‐based OLEDs.

## 2 Results and Discussion

### 2.1 Ground and Excited State Geometries

We begin by outlining the rationale for targeting the new Pt(II) complex used in this study, **Pt(8ppz)_2_**, which is guided by density functional theory (DFT) and quantum mechanical (QM) calculations that provide insights into the anticipated excited‐state geometry, Pt–Pt intermolecular distances, and their influence on modulating MMLCT emission characteristics. **Pt(8ppz)_2_** was designed as a structurally analogous control compound for an optimized MMLCT emitter, **Pt(fppz)_2_**. The Pt(II) coordination core is retained, while the peripheral substituents are changed from compact electron‐withdrawing ─CF_3_ groups in **Pt(fppz)_2_** to n‐octyl chains in **Pt(8ppz)_2_**. This modification was introduced deliberately to alter the preferred solid‐state packing and increase the Pt–Pt separation, thereby testing whether short‐range Pt–Pt interactions are required for efficient MMLCT formation. The design is motivated by alkylated organic semiconductors such as BTBT derivatives, where long alkyl chains can direct lamellar and herringbone packing through side‐chain interdigitation and zipper/fastener‐type interactions [29, 30]. Thus, the n‐octyl chain in Pt(8ppz)_2_ was used as a packing‐control element to perturb the local Pt–Pt fine structure. The ground‐state (S_0_) and excited‐state triplet (T_1_) geometries of the Pt complexes were optimized using DFT at the B3LYP‐D3/Gen1 level, incorporating Grimme's D3 dispersion corrections [31]. The same computational approach was employed for the molecular electrostatic potential (MESP) analysis, which provides a visual representation of the charge distribution within a molecule, indicating regions prone to electrophilic or nucleophilic interactions [32]. The optimized geometries are provided in Figure S1. In the S_0_ state, the calculated Pt–N_(pyridine)_ and Pt–N_(pyrazolate)_ bond lengths are 2.07 and 2.03 Å, respectively, for both **Pt(8ppz)_2_** and **Pt(fppz)_2_**. Upon excitation, the S_1_ states of both complexes exhibit a more pronounced metal–ligand interaction, as evidenced by shortened bond lengths, with the Pt–N_(pyrazolate)_ bonds displaying greater strengthening than the Pt–N_(pyridine)_ bonds. Figure 1a,b and Figure S2 present the MESP distributions mapped onto an isodensity surface of 0.01 a.u. for both complexes. The pyrazolate units in **Pt(8ppz)_2_** are comparatively more electron‐rich than those in **Pt(fppz)_2_**, owing to the electron‐donating nature of the n‐octyl substituents. Conversely, the pyridine units in **Pt(fppz)_2_** are more electron‐deficient than those in **Pt(8ppz)_2_**, attributable to the electron‐withdrawing ─CF_3_ groups. Complementary insights into the electron density distribution are provided by frontier molecular orbital (FMO) analysis. In both complexes, the highest occupied molecular orbital (HOMO) is primarily localized over the pyrazolate moieties and the metal center, whereas the lowest unoccupied molecular orbital (LUMO) is predominantly distributed over the pyridyl units and the metal center (see Figure S2). The HOMO exhibits a nodal plane at the Pt–N_(pyrazolate)_ bonding region, while the LUMO displays bonding interactions across all Pt–N linkages. In the S_1_ state, the single occupancy of both the HOMO and LUMO reduces the antibonding contribution at the Pt–N_(pyrazolate)_ bonds and enhances bonding interactions at all Pt–N sites, thereby rationalizing the observed bond strengthening relative to the S_0_ state. Notably, structural modification by replacing the electron‐withdrawing ─CF_3_ substituents in **Pt(fppz)_2_** with electron‐donating n‐octyl chains in **Pt(8ppz)_2_** results in a marked redistribution of the MESP. This alteration is anticipated to significantly influence the local Pt coordination environment and, consequently, modulate the excited‐state dynamics in neat films.

To probe intermolecular interactions, we extended our analysis from the monomeric species to their corresponding dimers. The geometries of the dimer complexes were optimized using the same level of theory employed for the corresponding monomer complexes, yielding Pt–Pt separations of 4.1 Å for **Pt(8ppz)_2_** and 3.5 Å for **Pt(fppz)_2_** as shown in Figure 1c,d. Notably, as confirmed by the EXAFS and GIWAXS results presented later, the ─CF_3_‐substituted **Pt(fppz)_2_** dimers adopt a stacked arrangement with a Pt–Pt distance of ≈3.4 Å, in excellent agreement with the optimized value. In contrast, the calculated Pt–Pt separation for **Pt(8ppz)_2_** is shorter by 1.4 Å compared to the experimental value of ≈5.5 Å. This deviation is expected because the long alkyl chains strongly influence molecular packing in the solid state, promoting interdigitation and thereby increasing the separation between adjacent Pt(II) units. The computed dimerization energies, Ed, are −41.7 kcal mol^−^^1^ for Pt(8ppz)_2_ and −36.8 kcal mol^−^^1^ for Pt(fppz)_2_, supporting the propensity of both complexes to form stable dimers.

### 2.2 Material Synthesis

**Pt(fppz)_2_** was synthesized following a previously reported procedure, while the alkylated derivative **Pt(8ppz)_2_** was prepared using a modified synthetic pathway [33, 34]. Full experimental and characterization data can be found in see Section S1, S2 and S4. Both Pt(II) complexes showed good stability in both solution and the solid state. However, while Pt(8ppz)_2_ has moderate solubility, Pt(fppz)_2_ has negligible solubility in common organic solvents at room temperature. Consequently, thermal gradient vacuum sublimation was utilized to produce high‐purity samples (see Figure S4). Material characterisation details can be found in Section S4, Figures S3–S5.

### 2.3 Crystal Packing and Pt Short‐Range Fine Structure

**FIGURE 2:**
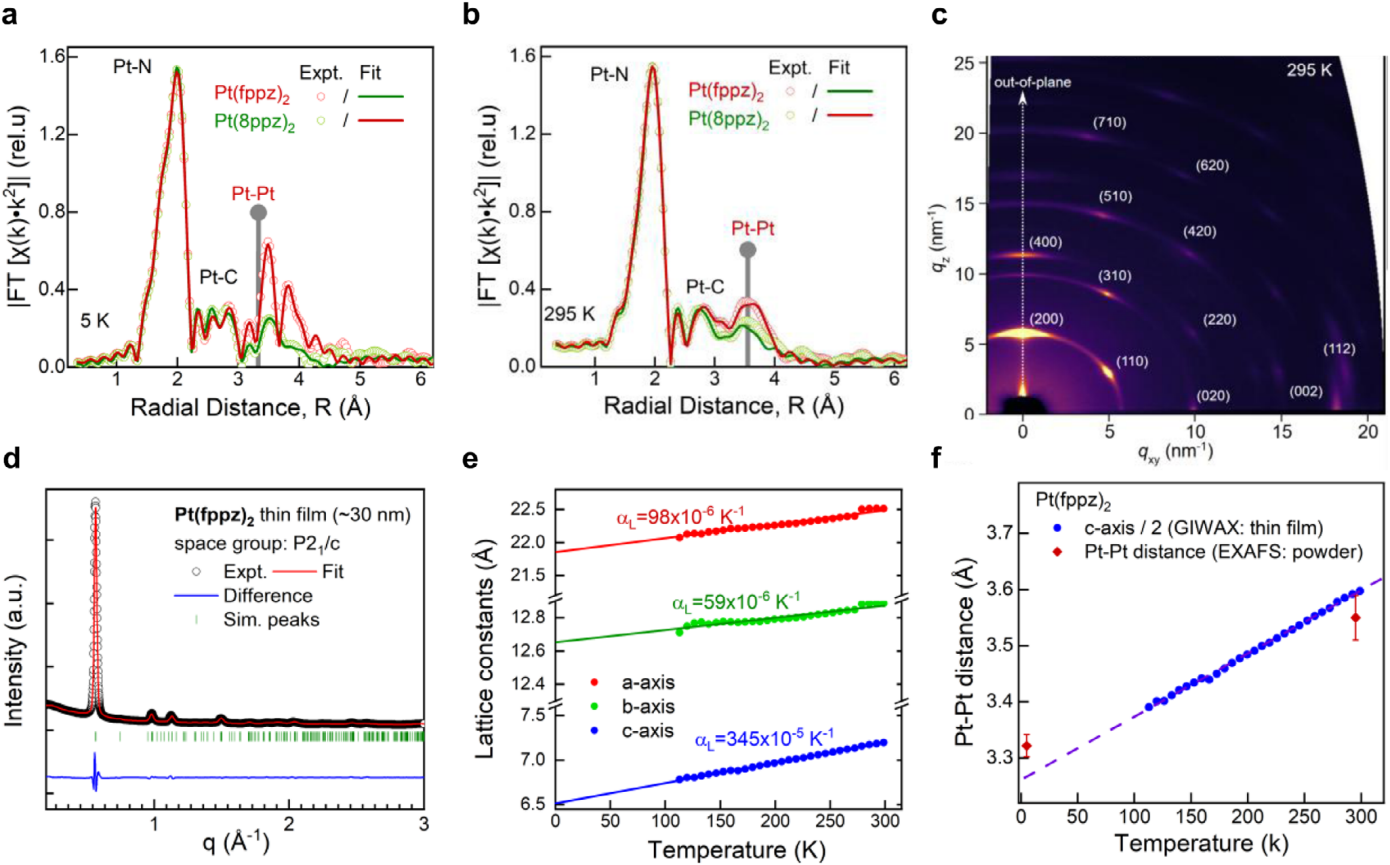
Fine structure of Pt(II) complexes. The phase‐corrected Fourier‐transform magnitude (k^2^‐weighted) of the EXAFS data (Figure S7) recorded from the powdered Pt complexes at 5 (a) and 295 K (b). The centre position of the extracted Pt–Pt scattering pair for the case of the **Pt(fppz)_2_** system is indicated by the vertical line. Experimental k^2^‐weighted *X*(k) EXAFS data (grey line) and the fits (coloured lines) corresponding to the different structural models are further shown in Figure S8. (c) Room‐temperature 2D GIWAXS pattern recorded from a highly‐oriented 30 nm‐thick **Pt(fppz)_2_** thin film at its critical angle (0.15°), with the assigned Bragg peaks and relative out‐of‐plane direction identified. For simplicity, this image does not account for the missing wedge. (d) Corresponding integrated GIWAXS scattering profile (q_xyz_) and structural refinement (Le Bail method). (e) Temperature‐dependence of the unit cell lattice parameters extracted from further low‐temperature GIWAXS measurements and subsequent refinements. (f) Comparison of the temperature‐dependent Pt–Pt distance in crystalline **Pt(fppz)_2_** determined from EXAFS fitting of the powdered samples and the distance inferred from evaluating thermal expansion along half of the *c*‐axis, resolved via structural refinements of the GIWAXS data. The dashed line captures the linear trend and is an aid for the eye.

The crystal structure of **Pt(8ppz)_2_** (Figure S6) determined from x‐ray crystallography shows perpendicular alignment of the alkyl chains relative to the pyrazole ring with both alkyl units interdigitating throughout the crystal structure. However, while there was strong alignment of **Pt(8ppz)_2_**, the complexes were in a slipped stack arrangement with short face‐to‐face distances of 3.31 Å and an extended Pt–Pt distance of 5.52 Å. The Pt coordination environments of **Pt(8ppz)_2_** and **Pt(fppz)_2_** were determined using extended x‐ray absorption fine structure (EXAFS) analysis at both cryogenic (5 K) and room temperature (295 K). The Fourier transforms (FT) of the EXAFS data are presented in Figure 2a, revealing large fine structure differences between these molecular crystals based on their known crystalline structures. Their common intramolecular bonding yields agreeable features below 3.1 Å. Beyond 3.1 Å, however, very different fine structure is resolved for the **Pt(fppz)_2_** sample at low temperature (5 K) due to the emergence of the Pt–Pt intermolecular scattering pairs (modelled to be 3.32 Å) between closely stacked molecules forming the crystals. We note that such a scattering feature is not resolved within the data recorded from **Pt(8ppz)_2_**, as the Pt–Pt distance is expected to exceed 5.5 Å and, thus, limits the sensitivity of the EXAFS probe. For the data recorded at a higher temperature (295 K), there exists a similar qualitative trend in the FT of the EXAFS (Figure 2a) for the two structures with the Pt–Pt scattering pair signal in the **Pt(fppz)_2_** crystal being expectedly elongated, broadened and less pronounced due to thermal‐induced effects. Comparing the coordination numbers (N) determined from the EXAFS fits (Table S1), there is a shift in the number of Pt–Pt molecular stacks determined at high‐ and low‐temperatures for the **Pt(fppz)_2_** sample. Specifically, at 5 K the coordination number is calculated to be N = 1.6 ± 0.2, corresponding approximately to an average of 4–10 coherently stacked molecules. At room temperature, the coordination number decreases to N = 1.2 ± 0.3, indicating only about 2–4 molecular stacks on average. This temperature‐dependent change in stack size reflects an increase in the degree of excited‐state delocalization at lower temperatures. Such behavior is in compelling agreement with the quantum mechanical predictions of Nakagaki et al., who demonstrated that low‐temperature crystal structures stabilizes the more extended ^3^MMLCT excited states, with ^3^[Pt]_4_ being more prevalent at low temperatures and ^3^[Pt]_3_ dominating at room temperature—thereby shifting the balance toward larger, more delocalized aggregates [35].

To evaluate the long‐range crystalline structure on the Pt(fppz)_2_ thin films, synchrotron grazing incidence wide‐angle scattering (GIWAXS) measurements were carried out between 80 and 295 K. A representative 2D GIWAXS pattern recorded from the Pt(fppz)2 thin film at room temperature is displayed in Figure 2b, along with the subsequent structural refinement of the azimuthally integrated scattering profiles in Figure 2c. The crystal is refined in the P21/c space group [a = 22.05(3) Å, b = 12.72(5) Å, c = 6.75(7) Å, α = γ = 90°, β = 94.9°], in agreement with reports previous reports [21]. Moreover, the film is found to be highly oriented, with the a‐axis pointing along the normal direction in a relatively narrow azimuthal distribution (Figure S9). In contrast, GIWAXS measurements of the evaporated **Pt(8ppz)_2_** film show only weak, broad diffuse scattering, indicating that the film is predominantly amorphous, with limited short‐range intermolecular correlations and no substantial long‐range crystalline order.

The inset of Figure 2c shows the relative thermal expansion of the lattice parameters heading toward liquid nitrogen temperature. The symmetry/phase and the orientation direction (Figure 2d) of the thin film crystal domains are preserved over this temperature range. Notably, there is a large inter‐molecular contraction along the ionic c‐axis, which might increase the intermolecular charge‐transfer character in thin‐film **Pt(fppz)_2_**. We note that that the rate of thermal expansion (α_L_ ≈35 × 10^−6^ K^−1^) along the *c*‐axis is significantly larger than the a‐ and b‐axes, and much larger than nominal organic materials (≈20 × 10^−6^ K^−1^). This suggests significant phonon anharmonicity along the *π*–*π*/Pt–Pt direction and explains the suppressed contraction close to 0 K determined using EXAFS (out of the range of T‐dependent GIWAX data), as phonon populations turns off, governed by Bose–Einstein statistics. Importantly, this also supports the large reduction in the Pt–Pt scattering pair intensity retrieved from the EXAFS data recorded at 300 K. These structural studies reveal that **Pt(fppz)_2_** adopts a more closely packed arrangement with shorter Pt–Pt separations compared to **Pt(8ppz)_2_**, which will be shown to have a direct impact on the excited‐state properties through the formation of the MMLCT state.

### 2.4 Steady State and Time Resolved Photophysics

**FIGURE 3:**
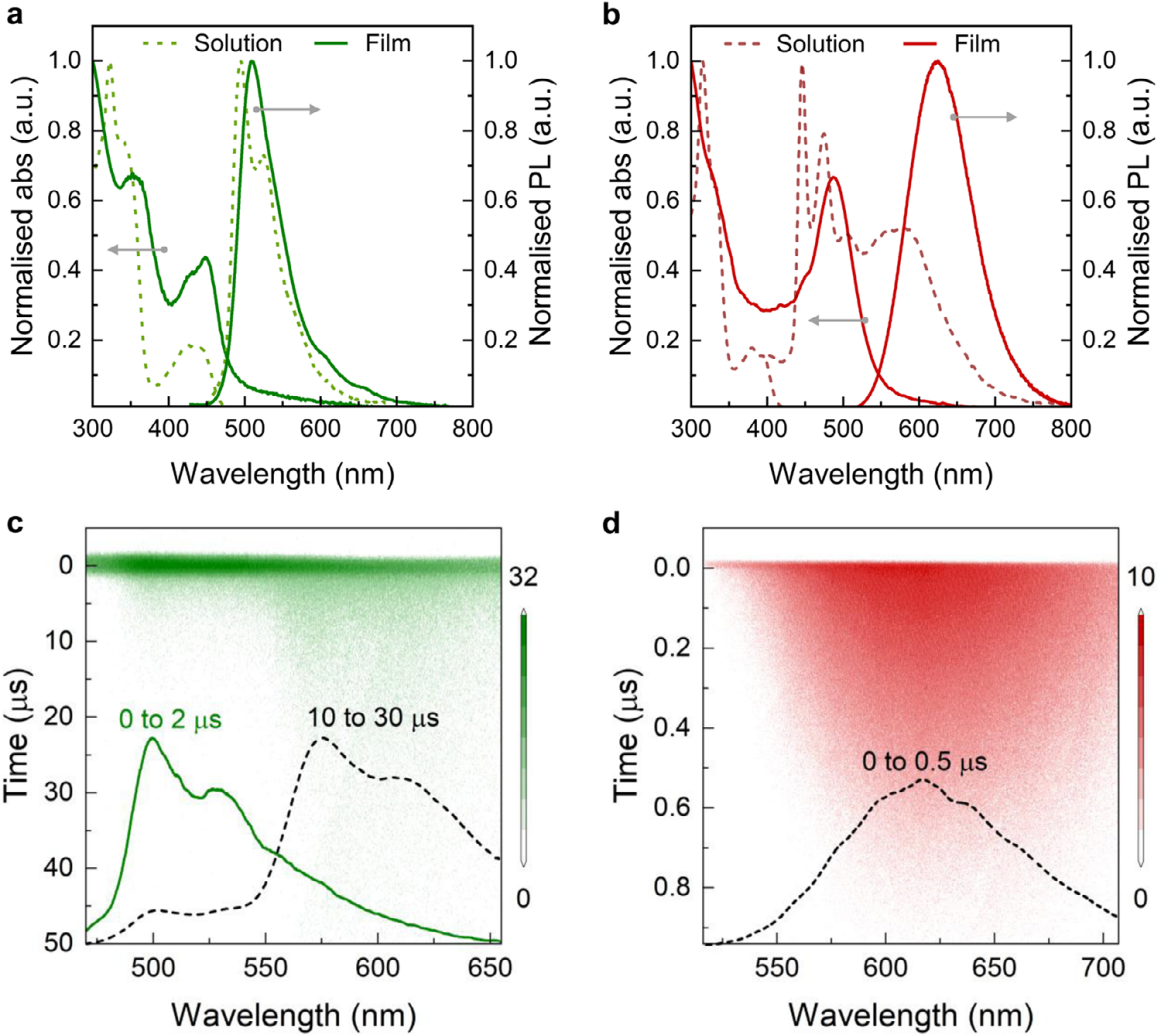
Photophysical properties of Pt(8ppz)_2_ and Pt(fppz)_2_ in solution and solid state. Absorption and photoluminescence (PL) spectra of (a) Pt(8ppz)_2_ and (b) Pt(fppz)_2_ in deoxygenated toluene solutions and in vacuum‐deposited thin films (50 nm). Pseudo–2D plots of time‐resolved PL (TR‐PL) spectra for (c) Pt(8ppz)_2_ and (d) Pt(fppz)_2_ neat films under 400 nm excitation. Time‐sliced spectra at selected delay intervals are shown for clarity.

The steady‐state photophysical properties of the Pt complexes were measured in toluene solution and vacuum‐deposited neat films as shown in Figure 3a,b. In toluene solutions, the absorption spectrum of the complexes can be divided into two main regions: a high‐energy region (<350 nm) with relatively high molar extinction and a weak absorption band at lower photon energies (390–460 nm for **Pt(8ppz)_2_** and 350–400 nm for **Pt(fppz)_2_**). Herein, the higher energy absorption can be attributed to the *π* to *π* ^*^ transitions of the ligands, while the longer wavelength absorption band can be assigned to the singlet metal‐to‐ligand charge transfer state (^1^MLCT) transition. Interestingly, both the absorption and PL spectra of **Pt(8ppz)_2_** in toluene were found to be red shifted by about 50 nm compared to **Pt(fppz)_2_**. The PL of both complexes were dominated by well‐resolved vibronic features; **Pt(fppz)_2_** exhibited an additional broad tail in the PL with a peak around 600 nm, which can be attribute to the aggregated states due poor solubility of **Pt(fppz)_2_** in common organic solvents. Together, the relatively large Stokes shift and large emission line width seen in **Pt(fppz)_2_** is an indicator of strong anharmonicity and carrier‐phonon coupling in the system, which is important for MMLCT state formation.

The PLQY of the two solution complexes were measured under ambient and deoxygenated conditions. Under deoxygenated conditions, **Pt(8ppz)_2_** showed a moderate PLQY of ≈31% with a mono‐exponential decay lifetime of ≈1.6 µs. The PLQY was found to be quenched to <5% under ambient conditions due to molecular oxygen, suggesting that the PL originates from the ^3^MLCT state. On the other hand, **Pt(fppz)_2_** exhibited significantly low PLQY (<1%) in solution along with a short decay lifetime of <10 ns. Furthermore, the PLQY and decay lifetime was found to be unaltered under ambient conditions (see Figure S10). This indicates the emission is from a weakly emissive ^1^MLCT state or consequently, the highly non‐radiative nature of the ^3^MLCT state.

Moving from solution to neat films, **Pt(8ppz)_2_** showed only a minor shift in the MLCT absorption band with a peak near 450 nm. The PL spectrum of **Pt(8ppz)_2_** also showed only a minor red shift of ≈15 nm with an additional weak emission at wavelengths >600 nm. Herein, the weak emission shoulder at longer wavelengths can be attributed to aggregate‐related intermolecular emission, which will be discussed in detail later (vide infra). Further, there was only a minor increase in PLQY of neat films (44%) compared to the deoxygenated toluene solution. In contrast, **Pt(fppz)_2_** showed a strong red shift in both absorption and PL. The origin of the strong absorption band (≈490 nm) and PL peak (620 nm) can be attributed to the MMLCT emission, resulting in close to unity PLQY for the **Pt(fppz)_2_** thin films. Next, we measured the PL decay lifetime for neat films of **Pt(8ppz)_2_** and **Pt(fppz)_2_** using time‐correlated single‐photo counting (TCSPC). **Pt(8ppz)_2_** exhibited a biexponential lifetime (Figure S11 and Table 1) with a short‐lived component of 0.7 µs (monitored at λ_PL_≈500 nm) and a long‐lived component of ≈8.6 µs as confirmed by PL decay kinetics in streak camera measurements discussed later. Here, the short‐lived feature can be attributed to isolated molecules, whereas the long‐lived component is assigned to delayed intermolecular ^3^LC/excimer‐like emission originating from aggregated species. Interestingly, **Pt(fppz)_2_** exhibited an apparent monoexponential decay with a lifetime of 370 ns (see Figure S11 and Table 1). The relatively fast PL decay of **Pt(fppz)_2_** results in a high radiative rate, originating from a high oscillator strength due to MMLCT transition and is explored in more detail below. To examine the possible presence of slow residual emissive components, long‐timescale wavelength‐dependent PL decay profiles were further recorded and are presented in Figure S12. **Pt(fppz)_2_** shows wavelength‐independent decay across the emission band, with an apparent lifetime of ≈370 ns and no significant long‐lived residual component within the measured time window of 50 µs. In contrast, **Pt(8ppz)_2_** exhibits wavelength‐dependent biexponential dynamics, with faster decay near 500 nm and a pronounced long‐lived component at 575–600 nm, consistent with delayed intermolecular ^3^LC/excimer‐like emission.

**TABLE 1:** Summary of photophysical properties of platinum complexes.

		λ_Abs_ [nm]	PLQY [%]	λ_PL_ [nm]	τ_av_ [µs]	*k*_r_ [10^5^ s^−1^]
**Pt(8ppz)_2_**	Solution	322, 426	31	495	1.6	1.84
	Neat Film	294, 355, 449	44	509	0.70, 8.6	6.31
**Pt(fppz)_2_**	Solution	315, 382	<1	447	< 0.01	−
	Neat Film	489	93	624	0.37	25.3

^a^ PL and PLQY measurements were performed using 325 nm excitation wavelength.

^b^ PL Lifetime measured were performed using 375 nm excitation wavelength

To further elucidate the underlying emission mechanism, time‐resolved photoluminescence (TR‐PL) studies are shown in Figure 3c,d. Initially, **Pt(8ppz)_2_** shows an emission centered at 520 nm right after excitation, characterized by a vibronic structure with a coupling frequency of 1280 cm^−1^ implied by the energy difference between the peaks of 0‐0 and 0–1 transitions. This supports the hypothesis that the emissive state is associated with the ring‐stretching vibration of the ligand, indicating ligand‐centered (LC, *π*–*π* ^*^) emission rather than charge‐transfer (CT) emission. This initial emission diminishes over approximately 1 µs, eventually shifting to a red‐shifted emission centered at 600 nm. The spectral shift implies that the shoulder around 600 nm in the steady‐state photoluminescence spectrum corresponds to this delayed emission component. The continued presence of vibronic structure suggests that the emission arises from an excimer state stemming from the LC state. In contrast, **Pt(fppz)_2_** exhibits a broad emission centered at 620 nm immediately following excitation. Although the emission peak undergoes a slight bathochromic shift over time, the overall spectral profile remains largely unchanged with a decay lifetime of approximately 300 ns, consistent with the TCSPC measurements. The minor bathochromic shifts are consistent with the findings of Chow et al., who attribute this behavior to the presence of multiple local minima in the electronic ground and excited states, resulting from subtle variations in interchain distances [36].

### 2.5 Ultrafast Dynamics of Excited‐States and Emission Mechanisms

**FIGURE 4:**
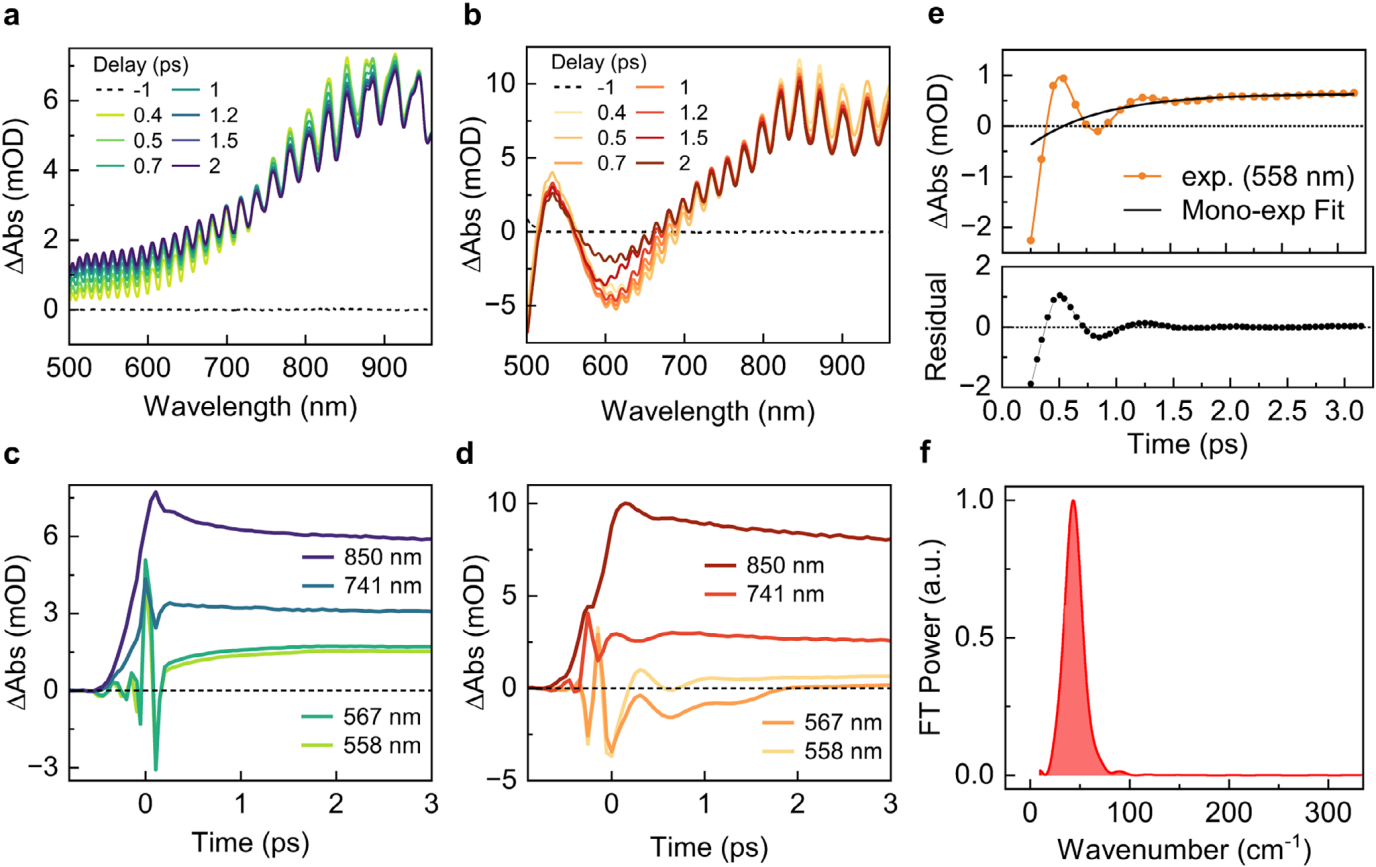
Ultrafast transient absorption (TA) dynamics of Pt(8ppz)_2_ and Pt(fppz)_2_ films. (a, b) Femtosecond TA spectra and (c, d) corresponding temporal profiles of (a, c) **Pt(8ppz)_2_** and (b, d) **Pt(fppz)_2_** neat films. (e) *Top*: Temporal profile of **Pt(fppz)_2_** at 558 nm (orange) with a monoexponential fit (black). *Bottom*: Residuals of the temporal profile from the fit. (f) Fourier‐transformed power spectrum of the oscillatory components.

To investigate the excited state dynamics and obtain direct evidence of MMLCT state formation, we conducted femtosecond transient absorption (fsTA) spectroscopy. Figure 4a,b present the fsTA spectra of **Pt(8ppz)_2_** and **Pt(fppz)_2_** neat films, respectively. For Pt(8ppz)_2_, we observed excited‐state absorption (ESA) centred at 900 nm, while **Pt(fppz)_2_** exhibited stimulated emission (SE) ≈620 nm and ESA near 820 nm. Both spectra displayed prominent fringe patterns due to interference, which do not affect the discussion of excited‐state dynamics. Figure 4c,d illustrate the time profiles of the TA signals at selected wavelengths. **Pt(8ppz)_2_** demonstrated monotonic decay or rise, whereas **Pt(fppz)_2_** exhibited oscillatory signals superimposed on the TA signals, in addition to coherent artifacts observed during excitation. These oscillatory components originated from nuclear wave packets in the excited state, attributed to coherent Pt–Pt stretching vibrations [37, 38, 39]. To analyze these oscillations, we subtracted the population component at 558 nm in the SE region using a single exponential function and performed fast Fourier transformation on the residual oscillatory signals. This analysis revealed an oscillation with a peak frequency centered at 45 cm^−1^ (see Figure 4e,f). We attribute this well‐defined, low‐frequency oscillation to Pt–Pt stretching associated with the MMLCT transition, providing strong evidence that **Pt(fppz)_2_** forms an MMLCT state, while **Pt(8ppz)_2_** does not.

These results, together with steady‐state and time‐resolved photoluminescence measurements, confirm that **Pt(8ppz)_2_** initially forms a metal‐to‐ligand charge transfer (MLCT) state upon excitation, which subsequently relaxes to a ligand‐centered (LC) state before ultimately emitting from an excimer state. In contrast, **Pt(fppz)_2_** directly accesses a metal–metal‐to‐ligand charge transfer (MMLCT) state immediately after excitation and emits from this state without intermediate relaxation, consistent with strongly anharmonic structural dynamics and coherent vibrational oscillations observed in the femtosecond transient absorption (fs‐TA) spectra.

### 2.6 Electroluminescence Properties

**FIGURE 5:**
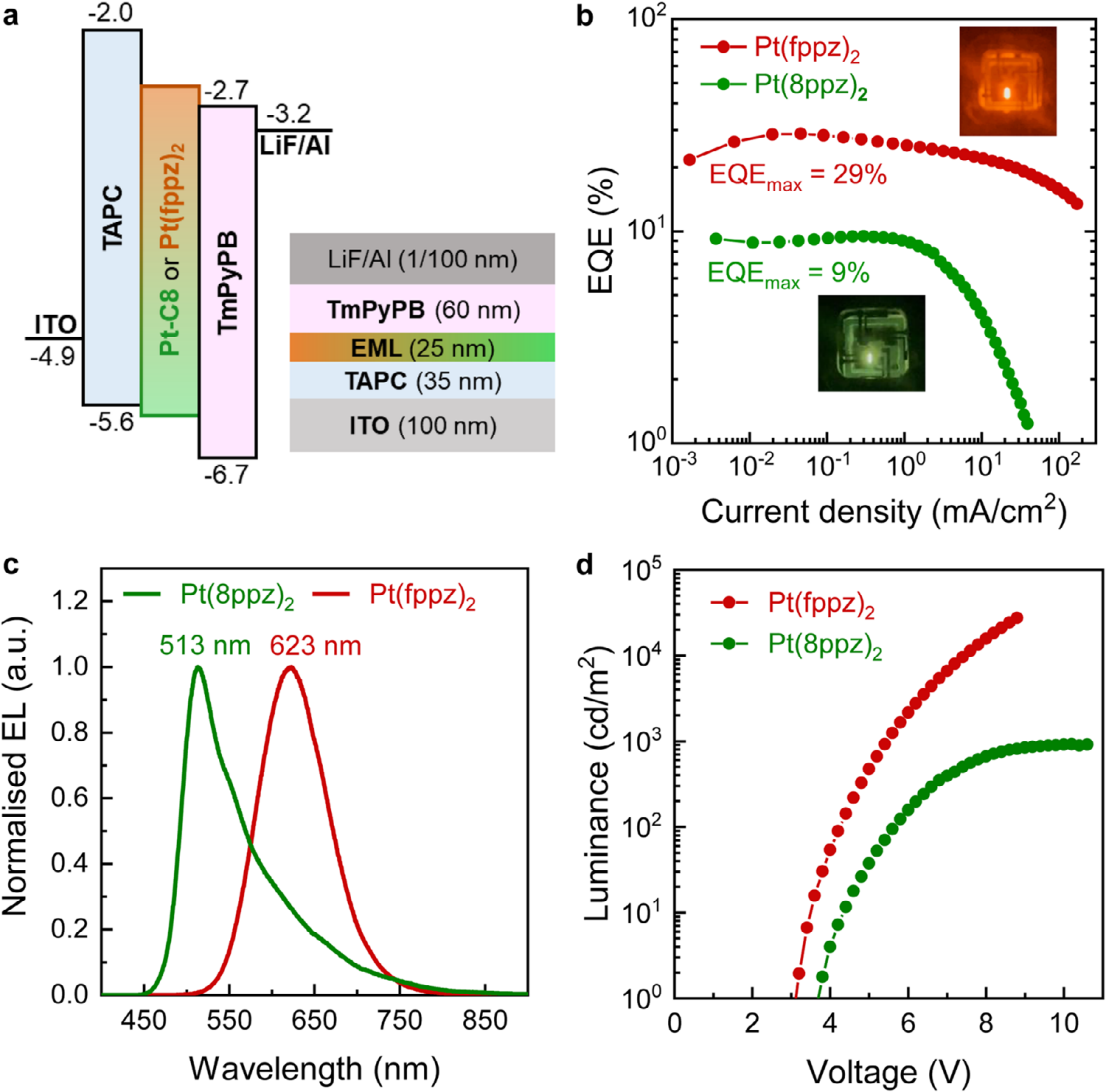
Electroluminescent characteristics of Pt(fppz)_2_ and Pt(8ppz)_2_ based OLEDs. (a) Energy level diagram and device architectures of OLEDs employing neat films of **Pt(fppz)_2_** (orange) and **Pt(8ppz)_2_** (green). (b) External quantum efficiency (EQE) as a function of current density for **Pt(fppz)_2_** and **Pt(8ppz)_2_** based devices (inset: photograph of the OLEDs operating at 8 V). (c) Electroluminescence (EL) spectra and (d) luminance–voltage (L–V) characteristics of the corresponding OLEDs.

To investigate the electroluminescence (EL) properties of Pt(fppz)_2_ and Pt(8ppz)_2_, neat‐film OLEDs were fabricated with device architecture: ITO (100 nm)/TAPC (35 nm)/**Pt(fppz)_2_** or **Pt(8ppz)_2_** (25 nm)/TmPyPB (60 nm)/LiF (1 nm)/Al (100 nm), where ITO, TAPC and TmPyPB are indium tin oxide, 4,4'‐cyclohexylidenebis[*N*, *N*‐bis(4‐methylphenyl)benzenamine] and 1,3,5‐tris(3‐pyridyl‐3‐phenyl)benzene, respectively (see Figure 5a for the device structures and their energy levels). Figure 5b depicts the EQE as a function of current density of the two OLEDs with their respective EL spectrum and luminance vs. voltage characteristics shown in Figure 5c,d, respectively. **Pt(fppz)_2_** devices showed a significant improvement in all the performance parameters, compared to **Pt(8ppz)_2_**‐based OLEDs. The max EQEs of Pt(fppz)_2_ and Pt(8ppz)_2_ devices reached 29% and 9%, respectively, as shown in Figure 5b. The enhancement in EQEs for **Pt(fppz)_2_** device can be attributed to better charge balance, high solid‐state PLQY (93%), and efficient out‐coupling efficiency of ≈31%, in complete agreement with the reduction of static disorder in **Pt(fppz)_2_** evaporated films as previously reported [21, 22], where the flat molecular geometry of **Pt(fppz)_2_** along with coherently stacked molecules provides efficient outcoupling in the forward direction. On the contrary, assuming the PLQY of 44% for **Pt(8ppz)_2_**, the light outcoupling efficiency was estimated to be close to 20%. The EL spectra of the two devices were found to closely align with the PL (see Figure 5c), suggesting a good confinement of the recombination zone within the EML and that the EL and PL originate from the same excited states [^3^LC and ^3^MMLCT states for **Pt(8ppz)_2_** and **Pt(fppz)_2_**, respectively]. Importantly, the **Pt(fppz)_2_**‐based OLED demonstrates a remarkably high EQE with minimal roll‐off, despite employing a relatively simple device architecture. Under identical device conditions, **Pt(8ppz)_2_** exhibits nearly a tenfold reduction in EQE at 1000 cd m^−^^2^ compared to its peak value, whereas **Pt(fppz)_2_** retains approximately 65% of its peak EQE even at a tenfold higher luminance of 10 000 cd m^−^^2^. This striking difference underscores the crucial role of the short excited‐state lifetime associated with the ^3^MMLCT states in **Pt(fppz)_2_**.

We employed the pulse operation technique to further evaluate the performance limit of OLEDs under high brightness and current densities. A short voltage pulse of 10 µs pulse width was used to minimize the Joule heating. Figure S13 shows the current–voltage‐luminance characteristics of OLEDs under pulsed and DC operations. The peak current densities >1 A/cm^2^ along with brightness values approaching 10^5^ cd m^−2^ were achieved in **Pt(fppz)_2_** OLED with a simplistic 3‐layer device structure. Furthermore, the EQE remains above 10% at 35 000 cd m^−^^2^ and exceeds 5% at 80 000 cd m^−^^2^, demonstrating excellent efficiency retention at high brightness levels. In contrast, **Pt(8ppz)_2_** OLED suffers a drastic EQE roll‐off at increased current densities/luminance. These performance metrics surpass those of state‐of‐the‐art phosphorescent OLEDs based on Ir(III) and Pt(II) emitters in terms of both EQE and brightness, while their roll‐off characteristics rival those of high‐performing fluorescent OLEDs; notably, the ability of our devices to sustain such high efficiency under elevated brightness directly underscores the role of Pt–Pt structural engineering in overcoming common roll‐off limitations.

To further probe the origin of electroluminescence, EL decays were collected and analyzed under pulsed voltage excitation. The pulse width was kept close to 500 ns, allowing the EL response to approach a quasi‐steady‐state before turn‐off, while the electrical fall time was below 5 ns, ensuring that the measured decay is not limited by the voltage pulse fall time or the RC of the device [40]. Figure S14 shows the voltage‐dependent EL decays for both OLEDs. For the applied voltage range between 7–20 V, the EL decays of the **Pt(fppz)_2_** OLED were much faster than those of the **Pt(8ppz)_2_** OLED, confirming the fast MMLCT and slower LC/MLCT emission, respectively. Interestingly both OLEDs showed two EL decay features, a short‐lived feature with a lifetime following similar decay dynamics as PL and a slow decay tail. The prompt component follows similar dynamics to the corresponding optical PL decay and is therefore assigned to radiative decay of the molecular excited state. In contrast, the delayed EL tail is not adequately described by a single exponential lifetime. To avoid complications from high‐current nonlinear processes, such as exciton–exciton annihilation and exciton–polaron interactions, the **Pt(fppz)_2_** decay analysis was performed for the 7 V trace, which is the lowest‐voltage condition providing sufficient EL signal for reliable fitting. As shown in Figure S15, the early‐time decay is approximated by an exponential component, whereas the delayed tail follows a power‐law dependence. This behaviour indicates dispersive delayed recombination arising from a distribution of carrier recombination times in the OLED stack, rather than an additional intrinsic emissive state of **Pt(fppz)_2_** [41]. Such non‐exponential recombination dynamics are consistent with previous reports on disordered organic semiconductors, where energetic disorder, carrier relaxation within the density of states, trapping/release processes, and spatially inhomogeneous transport can broaden transient recombination dynamics [42, 43, 44]. Therefore, the TREL results support the conclusion that Pt(fppz)_2_ possesses a fast intrinsic ^3^MMLCT emissive decay, while the slower electrical tail originates primarily from delayed device‐level recombination. Hole‐only devices with the structure ITO/MoO_x_/Pt complex/MoO_x_/Au further show that Pt(fppz)_2_ exhibits an approximately threefold higher hole mobility than Pt(8ppz)_2_, consistent with more efficient intermolecular charge transport in the compactly packed Pt(fppz)_2_ film (Figure S16). Together, these results highlight that deliberate molecular design and precise control of Pt–Pt interactions provide the fundamental origin of enhanced efficiency and suppressed roll‐off, establishing Pt–Pt engineering as a powerful strategy for high‐performance OLEDs.

## 3 Conclusion

In conclusion, this study establishes a clear structure–property–performance relationship in square‐planar Pt(II) complexes, demonstrating that fine‐tuning short‐range Pt–Pt interactions is essential for optimizing OLED efficiency and stability. Structural analyses using temperature‐dependent EXAFS and GIWAXS reveal that **Pt(fppz)_2_** adopts a highly ordered packing motif with short Pt–Pt distances (3.32 Å), in contrast to the disordered, loosely packed **Pt(8ppz)_2_** (Pt–Pt > 5.5 Å). These structural differences directly influence excited‐state dynamics: **Pt(fppz)_2_** supports fast, efficient MMLCT emission (τ = 0.37 µs, near‐unity PLQY), accompanied by coherent Pt–Pt vibrational motion detected via fs‐TA, while **Pt(8ppz)_2_** exhibits inefficient, long‐lived excimer emission originating from LC excited state. These different photophysical behaviors translate into different device performance metrics, with **Pt(fppz)_2_** OLEDs achieving peak EQEs ≈29% and maintaining 23% at 5000 cd m^−^^2^ with minimal roll‐off. In contrast, Pt(8ppz)_2_ devices suffer from significant efficiency losses with pronounced roll‐off and limited brightness. These findings underscore the critical role of short‐range Pt–Pt interactions in enabling MMLCT emission and offer a broadly applicable strategy for designing high‐performance OLED materials with enhanced brightness and operational robustness.

## 4 Experimental

### 4.1 Synthesis and Characterization

All commercial reagents and chemicals were used as received unless otherwise noted. Column chromatography was completed using Merck LC60A 40‐30 silica gel, or Merck activated (neutral) Brockmann type I aluminium oxide with solvent ratios reported by volume. Melting points (mp) were measured in a glass capillary on a BÜCHI Melting Point B‐545 with a heating rate of 5°C min^−1^ and were quoted from the point of first melt to completely melted. Thermal gravimetric analysis (TGA) was carried out on a Perkin Elmer STA 6000 with a scan rate 10°C min^−1^ under continuous nitrogen flow, and the TGA decomposition temperature was quoted at 5% weight loss. Differential scanning calorimetry (DSC) was used to investigate thermal properties with heating and cooling rates of 50, 100 and 200°C min^−1^ under a nitrogen atmosphere with a Perkin Elmer Diamond DSC. High‐resolution mass spectrometry (HRMS) were recorded on an Orbitrap Elite MS in positive mode. Elemental analysis was completed at The University of Queensland. CreaPhys DSU05 was used for vacuum sublimation. Details of the material synthesis and characterization can be found in the Supporting Information.

### 4.2 Electrochemical Measurements

Electrochemistry was performed on an Epsilon C3 BAS electrochemistry station using a glassy carbon working electrode, a 0.1 m AgNO_3_ acetonitrile solution reference electrode, and a platinum counter electrode with a scan rate of 100 mV s^−1^. All measurements were completed at room temperature using 0.1 m tetra‐*n*‐butylammonium perchlorate as the electrolyte. All solutions were purged with argon where sample concentration was approximately 1 mm in dichloromethane (distilled from calcium hydride) and doubly distilled tetrahydrofuran (from sodium and then lithium aluminium hydride) for oxidation and reduction, respectively. For all measurements, the ferrocenium/ferrocene (Fc^+^/Fc) couple was used as the standard and in all cases several scans were completed to confirm the chemical reversibility of the redox processes.

### 4.3 Photophysical Measurements

UV–vis absorption spectra were recorded on a Varian Cary‐5000 UV–Vis spectrophotometer in 10 × 10 mm quartz cuvettes. Fluorescence spectra were measured using FS5 fluorescence spectrometer (Edinburgh Instruments) using a xenon lamp as the excitation source. Solution PLQYs were determined by the relative method with quinine sulphate as the reference (0.1 M in H_2_SO_4,_ PLQY = 55%). All solutions were prepared in spectroscopic grade toluene with a fixed optical density of ≈0.1 at the excitation wavelength of 350 nm. Solid‐state PLQY measurements were performed using a calibrated integrating sphere [45]. All the samples were excited at 325 nm using HeCd Laser (IK series, Kimmon). TCSPC measurements were performed using FS5 fluorescence spectrometer (Edinburgh Instruments) with a laser diode (375 nm) as the excitation source, and emission was monitored at the peak of the fluorescence spectrum.

### 4.4 Time‐Resolved Photoluminescence

Time‐resolved photoluminescence (TR‐PL) measurements were conducted using a polychromator and a streak camera system (Hamamatsu C4780) with a time resolution of less than 30 ps, synchronized with a Ti: sapphire amplifier. The excitation source (wavelength: 400 nm, fluence at sample position <1.5 mJ/cm^−2^) was second harmonic generation light, which was generated from the output of a Ti: sapphire regenerative amplifier (Spectra‐Physics Spitfire Ace, pulse duration: 120 fs, repetition rate: 1 kHz, pulse energy: 3.6 mJ pulse^−1^, and central wavelength: 800 nm) that was seeded by a Ti: sapphire femtosecond mode‐locked laser (Spectra‐Physics, Tsunami).

### 4.5 Femtosecond Transient Absorption Spectroscopy

Femtosecond transient absorption (fsTA) measurements were conducted using a custom‐built setup employing the pump‐probe method. The light source consisted of a Yb: KGW regenerative amplifier system (Light Conversion, PHAROS) with the following specifications: 190 fs pulse duration, 1 kHz repetition rate, 1 mJ pulse^−1^ energy, and 1030 nm wavelength. The amplifier output was split into two beams for generating the pump and probe pulses. The Pump beam was frequency‐chopped by half relative to the probe. Sample excitation was achieved using a 400 nm pump pulse, obtained through second harmonic generation of the output from an optical parametric amplifier system. A 3 mm‐thick sapphire crystal was utilized to generate the broadband probe pulse (500–950 nm). The time delay between pump and probe pulses was controlled by a computer‐operated mechanical delay stage. After passing through the sample films, the probe pulse was dispersed by a polychromator (JASCO, CT‐10, 300 grooves 500 nm^−1^), and the resulting spectra were recorded using a multichannel detection system equipped with a CMOS sensor (UNISOKU, USP‐PSMM‐NP). Data analysis was performed using a custom‐developed Python‐based program.

### 4.6 OLED Fabrication and Characterization

The steady‐state and transient EL measurements were performed using instrumentation described in our previous works [46, 47]. Transient EL decays were obtained by using a PMT (Hamamatsu H10721‐20) connected to a digital oscilloscope (Teledyne LeRoy, 2 GHz). An AVTECH pulse generator (AVX1011‐B1‐B) with a short rise and fall time of <5 ns was used as the electrical excitation source for the transient EL measurements.

### 4.7 Extended X‐Ray Absorption Fine Structure (EXAFS) Experiments

Pt L3‐edge x‐ray absorption spectra (XAS) were recorded at the XAS beamline 12‐ID at the Australian Synchrotron, ANSTO in Clayton, Victoria. The excitation energy was selected using a double‐crystal Si (111) monochromator with focusing optics to minimize harmonic content. Energy calibration of the monochromator was performed at the Pt−L3 absorption edge using an inline Pt metal foil (first maximum of the first derivative at 11564.18 eV). The energy of the absorption spectra was calibrated by measuring the XANES of a reference Pt foil provided by the synchrotron beamline, with the Pt foil's E_0_ set to its reported [48] value of 11562.76 eV.

Powder samples were pelletized to form compact 7 mm disks. All acquisitions were performed in transmission mode at both 5 and 300 K. Short, repeat XAS scans of representative samples were performed to confirm that the materials were sufficiently stable under at least 15 min of x‐ray exposure prior to acquisitions. Samples were measured in a slew‐scanning move with varying energy intervals over the pre‐edge (5 eV) and the XANES region (0.25), with 0.035 intervals in k‐space over the EXAFS. At low‐k, an integration time of 100 ms was employed per interval, with longer integration times weighted toward high‐k portions of the spectrum, up to a maximum value of 18 in k‐space (max of 1000 ms).

### 4.8 EXAFS Analysis

Multiple scans were collected at different sample positions. Data were pre‐processed using Athena program for scan averaging, background subtraction, edge‐height normalization, and rebinning [49]. To normalize the energy spectra and remove background, the pre‐edge range was set to between −180 and −50 eV, and the normalization range was set from 150 to 1230 eV post‐edge. The normalization order was set to 3. The k‐weight was set to 2 for all presented EXAFS data, and depending on the temperature, the k‐range for forward Fourier transform was set from 2 to 13 (300 K) or 2 to 16 (5 K).

EXAFS fitting was conducted in ARTEMIS, part of the IFEFFIT software package [49]. The scattering paths used to model the data were derived from the structure of Pt(fppz)_2_ crystal described by Kim et al., and from a chemical information file (CIF) file attained via personal communication with the authors [21], and the Pt(8ppz)_2_ single crystal structure measured from x‐ray diffraction (XRD) studies. Using these two input structures to derive the scattering paths, a common organic intramolecular fine structure was used to account for dominant EXAFS signals arising an R fitting window of 1.5 to 4.2 Å for both Pt(II) complexes. The inclusion of additional intermolecular scattering pairs (>3.3 Å) were used to well‐describe the observed fine structural differences in this regime; namely, the relatively strong signal arising from a Pt–Pt scattering pair (∼3.4 Å) in the case of crystalline Pt(fppz)_2_. See Figure S6 to visualize. Global bond expansion parameters were used to account for correlated variations in the intramolecular and intermolecular distances, based on the input paths generated by the CIF files in ARTEMIS. To reduce the number of variables further, terms used to model the Debye–Waller factors were shared across colinear multiple scattering paths. The data were ultimately fit using 28 variables and 39 independent points, resulting in reasonable R‐factor values (Table S1). The fitting was conducted in R‐space using multiple k‐weightings, with k = 1, 2 and 3. Errors of individual fit parameters were determined using ARTEMIS to take into account the correlations between parameters, and known parameters without error estimates were fixed during the fit.

### 4.9 Synchrotron‐Based Grazing Incidence Wide Angle X‐Ray Scattering (GIWAXS)

GIWAXS data were recorded at the NCD‐SWEET beamline at the ALBA synchrotron (Cerdanyola del Vallès, Spain): Monochromatic (λ = 0.9574 Å) x‐ray beam of 80 × 30 µm^2^ [H × V], using a Si (111) channel cut monochromator, which was collimated using an array of Be collimating lenses. The scattered signal was recorded using a Rayonix LX255‐HS area detector placed at 229.4 mm from the sample position. Detector tilts and sample‐to‐detector distance were calculated using Cr_2_O_3_ as a calibrant. GIWAXS frames were recorded at incident angles (α_i_) between 0° and 0.16°, remaining in the surface‐sensitive evanescent regime of scattering and avoiding penetration into the substrate. The critical angle of the 30 nm‐thick Pt(fppz)_2_ thin film was calculated to be 0.15°, with all subsequent analysis taking place at this angle. A continuous flow of N_2_ gas over the sample was employed during the measurements to ensure the sample remained in a chemically inert atmosphere during irradiation, and the temperature was varied using a nitrogen‐cooled Linkam stage. All collected 2D images were azimuthally integrated using PyFAI and processed using a custom Python routine [50].

## Author Contributions

Conceptualization was carried out by S.‐C.L. and E.B.N. Data curation and formal analysis were performed by A.S., S.K.M., I.G., J.A.S., M.H., C.M., K.M., R.J.L., E.S., P.K.R., C.H.S., and T.E. Methodology, experimental design, validation, and investigation were conducted by A.S., J.A.S., K.M., K.O., C.H.S., E.B.N., and S.‐C.L. Project administration, funding acquisition, and resources were handled by K.O., J.A.S., S.‐C.L., C.A., and E.B.N. Writing – original draft was carried out by A.S., J.A.S., and E.B.N., while Writing – review and editing were performed by all authors. All authors have read and approved the final manuscript.

## Conflicts of Interest

The authors declare no conflicts of interest.

## Supporting information




**Supporting File**: advs77325‐sup‐0001‐SuppMat.pdf

## Data Availability

The data that support the findings of this study are available from the corresponding author upon reasonable request.
