# Supplementary material for: Origin of Enhanced Efficiency and Reduced Roll‐Off in Organic Light‐Emitting Diodes Based on Pt(II) Complexes
Source: Adv Sci (Weinh). 2026 Aug 24;13(63):e77325. doi: 10.1002/advs.77325 (PMC13502274; doi:10.1002/advs.77325)
Supplement: Supplementary file 1 [file ADVS-13-e77325-s001.pdf]

Supporting Information

Origin of Enhanced Efficiency and Reduced Roll-Off in Organic Light-Emitting Diodes Based on Pt(II) Complexes

Atul Shukla,[1] Sarah K. M. McGregor,[2] Julian A. Steele,[1,3*] Innes K. Gale,[1,2] Kiyoshi Miyata,[4] Monirul Hasan, [1] Christian McDonald,[1] Romain J. Lepage,[2] Eduardo Solano,[5] Pilankatta K. Ramya,[6] Cherumuttathu H. Suresh,[6,7] Takumi Ehara,[4] Chihaya Adachi,[8] Ken Onda, [4*] Shih-Chun Lo,[2*] Ebinazar B. Namdas[1*]*

[1] School of Mathematics and Physics, The University of Queensland, Brisbane, QLD 4072, Australia

[2] School of Chemistry and Molecular Biosciences, The University of Queensland, Brisbane, QLD 4072, Australia

[3] Australian Institute for Bioengineering and Nanotechnology, The University of Queensland, Brisbane, Queensland 4072, Australia

[4] Department of Chemistry, Kyushu University, 744 Motooka, Nishi, Fukuoka 819-0395, Japan

[5] NCD-SWEET Beamline, ALBA Synchrotron Light Source, Cerdanyola del Vallés, 08290, Barcelona, Spain

[6] Chemical Sciences and Technology Division, CSIR-National Institute for Interdisciplinary Science and Technology, Thiruvananthapuram, Kerala, 695019, India

[7]Srinivasa Ramanujan Institute for Basic Sciences, Kottayam, Kerala, 686501, India

[8] Center for Organic Photonics and Electronics Research (OPERA), Kyushu University, 744 Motooka, Nishi, Fukuoka 819-0395, Japan

*Correspondence to: Ebinazar B. Namdas (e.namdas@uq.edu.au),

Shih-Chun Lo (s.lo@uq.edu.au),

Ken Onda (konda@chem.kyushu-univ.jp)

Julian A. Steele (julian.steele@uq.edu.au)

Atul Shukla (a.shukla@uq.edu.au)

Table of Contents

Section S1. General Experimental..S3.
Section S2. Material Synthesis..S4.
Pt(8ppz)$_2$..S4.
Pt(fppz)$_2$ ....S5.
Section S3. DFT and TD-DFT calculations ....S6.
Figure S1. Optimized geometries of S_0, S_1 and T_1 states of **Pt(8ppz)$_2$** and **Pt(fppz)$_2$**…S6.
Figure S2. MESP distribution of **Pt(8ppz)$_2$** and **Pt(fppz)$_2$**…......................S7.
Section S4. Material properties ....S7.
Thermal Properties..S7.
Figure S3. TGA and DSC traces of **Pt(8ppz)$_2$** and **Pt(fppz)$_2$**……………………S8.
Figure S4. Image of an example purification of **Pt(fppz)$_2$** by vacuum sublimation...........S8.
Electrochemical Properties..S9.
Figure S5. CV and DPV of **Pt(8ppz)$_2$** in solution…………………......S9.
Section S5. Molecular packing in single crystals and in thin films……………………S10.
Figure S6. Crystal structure of of **Pt(8ppz)$_2$** ……………………………S10.
Figure S7. Normalized EXAFS spectra of **Pt(fppz)$_2$** and **Pt(8ppz)$_2$** ……………………S11.
Figure S8. Comparison of common and unique intramolecular fine structures.................S11.
Table S1. EXAFS fitting parameters at 50 and 295 K..S12.
Figure S9. Scattering planes (200) and the relative angle illustration...............................S13.
Section S6: Photophysics Properties…………………………………………..S14.
Figure S10. Normalized solution PL of Pt(fppz)$_2$..S14.
Figure S11. PL decay of evaporated neat films for **Pt(fppz)$_2$** and **Pt(8ppz)$_2$**....................S14.
Figure S12. Wavelength dependent PL decay for **Pt(fppz)$_2$** and **Pt(8ppz)$_2$**......................S15.
Section S7: OLED Characteristics…………………………………………….S16.
Figure S13. Performance of **Pt(fppz)$_2$** and **Pt(8ppz)$_2$** based OLEDs................................S16.
Figure S14. EL decays of **Pt(fppz)$_2$** and **Pt(8ppz)$_2$** based OLEDs....................................S17.
Figure S15. EL decay Fits of **Pt(fppz)$_2$** based OLEDs at 7 V..S17.
Figure S16. Space-charge-limited-current measurments...S18.
Section S8: NMR and Mass Spectra..S18.
Figure S17. ^{1}H NMR (500 MHz, $CDCl_3$) of **Pt(8ppz)$_2$**..S18.
Figure S18. ^{13}C NMR (126 MHz, $CDCl_3$) of **Pt(8ppz)$_2$**...S19.
Figure S19. HRMS of N **Pt(8ppz)$_2$**..S19.
Figure S20. ^{1}H NMR (400 MHz, acetone-d_6) of **Pt(fppz)$_2$**...S20.
Section S9: Crystal Structure Coordinate ……………………………………S21.
Section S10: References…………………………………………………S24.

Section S1. General Experimental

All ^{1}H and ^{13}C NMR spectra were recorded using Bruker Avance 400 or 500 spectrometers in $CDCl_3$ or acetone-d_6. All chemical shifts (δ) were reported in parts per million (ppm) and referenced to the residual solvent peak, *i.e.*, δ 7.26 ppm ($CHCl_3$ for ^{1}H), 77.00 ppm ($CDCl_3$ for ^{13}C), and 2.05 ppm (acetone-d_6 for ^{1}H). Multiplicities were reported as singlet (s), doublet (d), and triplet (t); Py-H = pyridyl-H; Pz-H = pyrazol H. All coupling constants (*J*) were quoted in Hertz (Hz) and rounded to the nearest 0.5 Hz. HRMS were recorded on an Orbitrap Elite MS in positive mode. Source parameters included a heater temperature at 50 °C; sheath gas flow 6; ISV at 3.5 kV and S-lens at 60%, ion transfer tube temperature at 320 °C. Data was analysed using the on-board Xcalibur software. Melting points (mp) were measured in a glass capillary on a BÜCHI Melting Point B-545 with a heating rate of 5 °C min^{-1} and were quoted from the point of first melt to completely melted. Thermal gravimetric analysis (TGA) was carried out on a Perkin Elmer STA 6000 with scan rate 10 °C min^{-1} under continuous nitrogen flow and the TGA temperature was quoted at 5% weight loss as the decomposition temperature. Differential scanning calorimetry (DSC) was used to investigate thermal properties with heating and cooling rates between 50 °C min^{-1} and 200 °C min^{-1} under a nitrogen atmosphere with a Perkin Elmer Diamond DSC. Merck aluminium plates coated with 60 F254 silica gel were used for thin layer chromatography (TLC) and visualised using 254 nm (short wave) and 365 nm (long wave) light. Column chromatography was carried out using Merck LC60A 40-30 silica gel or Merck activated (neutral) Brockmann type I aluminium oxide.

Section S2. Material Synthesis

Pt(8ppz)$_2$

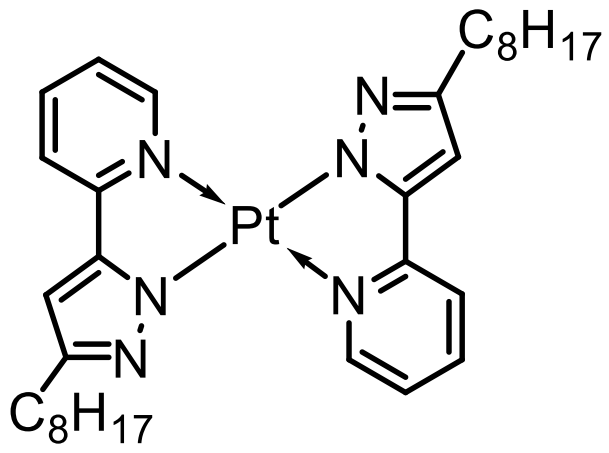

1,2-Dimethoxyethane (DME, 6 mL) was added to a mixture of Pt(DMSO)$_2$Cl$_2$ (266 mg, 0.63 mmol), Na_2CO_3 (2.06 g, 19.4 mmol), and ligand 8ppzH (1.00 g, 3.89 mmol). The mixture was heated at reflux under argon for 16 h. The mixture was cooled to room temperature. Solvent was removed under vacuum and the residue was taken up in dichloromethane (100 mL), and washed with water (3 × 100 mL), dried over anhydrous Na_2SO_4 and filtered. The filtrate was collected and the solvent was evaporated. The obtained crude product was purified by column chromatography over silica using dichloromethane and *n*-hexane (3:1) as the eluent, followed by recrystallization from a mixed solution of dichloromethane and *n*-hexane to give a pale yellow crystalline solid (279 mg, 62%). mp: 251–252 °C; T_{sub} = 210 °C at 1 × 10^{-6} mbar (amorphous); $T_{d(5\%)}$ = 385 °C; ^{1}H NMR (500 MHz, $CDCl_3$): δ 0.89 (t, *J* = 7.5, 6H, Me), 1.22–1.53 (m, 20H, CH_2), 1.72–1.82 (m, 4H, CH_2), 2.79 (t, *J* = 7.5, 4H, CH_2), 6.46 (s, 2H, Pz-H), 7.23 (ddd, *J* = 7.5, 6.0 &1.5, 2H, Py-H), 7.56 (ddd, *J* = 8.0, 1.5 & 1.0, 2H, Py-H), 7.85 (td, *J* = 7.7 & 1.5, 2H, Py-H), 10.68–10.84 (m, 2H, Py-H). ^{13}C NMR (126 MHz, $CDCl_3$): δ 14.13, 22.70, 28.93, 29.35, 29.57, 29.65, 30.14, 31.95, 101.79, 117.86, 120.84, 138.88, 149.80, 152.29, 152.92, 155.28. HRMS (ESI) m/z: $[M+H]^+$ Calcd for $C_{32}H_{45}N_6Pt$ 708.3348; Found 708.3336. Anal. Calcd for $C_{32}H_{44}N_6Pt$: C, 54.3; H, 6.3; N, 11.9. Found: C, 54.6; H, 6.1; N, 11.8.

Pt(fppz)$_2$

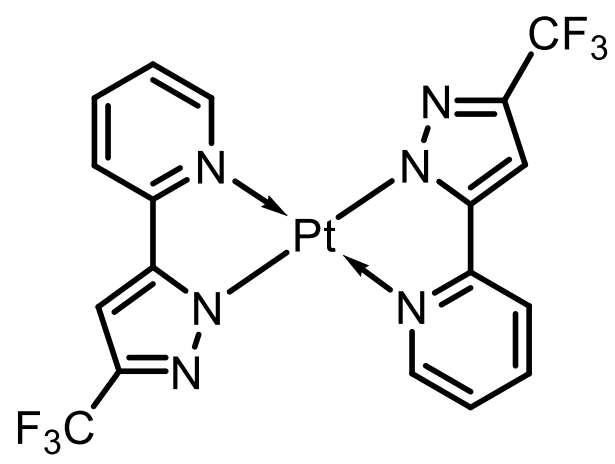

Ligand fppzH (565 mg, 2.65 mmol) was added to a Schlenk flask containing K_2PtCl_4 (505 mg, 1.21 mmol), ethanol (45 mL) and water (15 mL). The mixture was evacuated and backfilled with argon six times before being heated in an oil bath at 80 °C under argon for 22 hours, during which the initially red transparent solution became cloudy with significant orange precipitate. The solution was cooled to room temperature, and the solids were collected by vacuum filtration, washed with water, ethanol, dichloromethane, and diethyl ether. The brown powder (460 mg) was sonicated in a dichloromethane and ethanol mixture (1:3, 20 mL), then centrifuged before decanting off the mother liquor. Finally, the solids were suspended in acetonitrile and collected by vacuum filtration, washed with excess acetonitrile to remove the dark soluble material. The resulting poorly soluble solids were dried to give Pt(fppz)$_2$ as a bright orange powder (270 mg, 36%). The material can be further purified by fractional vacuum sublimation at 170 °C (at 4×10^{-7} mbar) at a rate of 0.15 mg h^{-1}, collecting the 100–115 °C fractions. mp: >300 °C; T_{sub} = 165 °C at 4×10^{-7} mbar (amorphous); $T_{d(5\%)}/T_{sub}$ under $N_{2(g)}$ = 347 °C; ^{1}H NMR (400 MHz, acetone-d$_6$) δ 7.11 (s, 2H, Pz-H), 7.51 (ddd, J = 7.5, 6.0 & 1.5, 2H, Py-H), 7.98 (ddd, J = 8.0, 1.5 & 1.0, 2H, Py-H), 8.22 (td, J = 7.5 & 1.5, 2H, Py-H), 10.24–10.47 (m, 2H, Py-H). ^{19}F NMR (376 MHz, acetone-d$_6$) δ -61.3 (s, CF_3). Anal. Calcd for $C_{18}H_{10}F_6N_6Pt$: C, 34.9; H, 1.6; N, 13.6. Found: C, 35.1; H, 1.5; N, 13.4.

Section S3. DFT and TD-DFT calculations

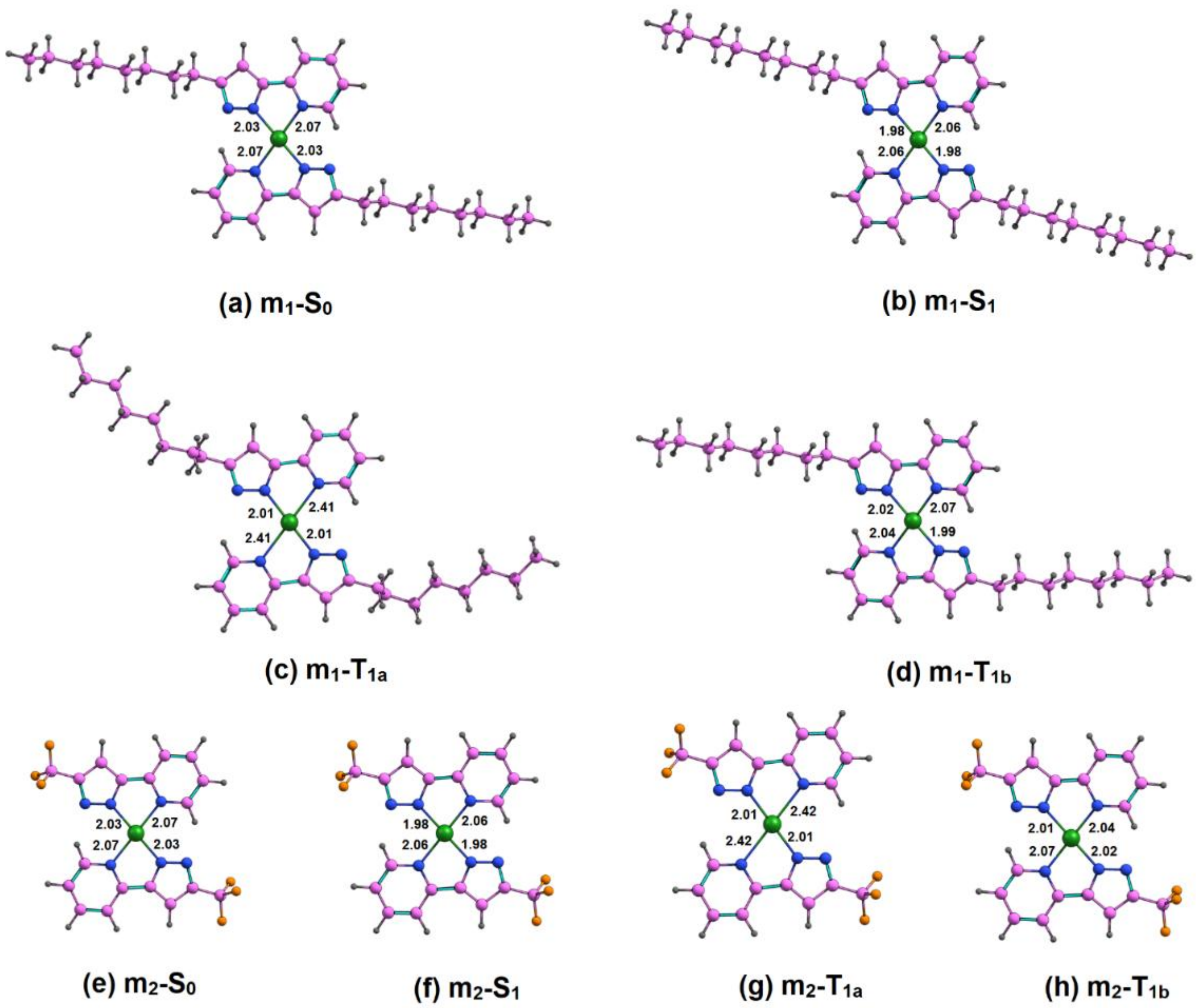

Figure S1. Optimized geometries of S_0, S_1 and T_1 states of Pt(8ppz)$_2$ (denoted as m_1) and Pt(fppz)$_2$ (denoted as m_2) with Pt-N bond lengths in Å.

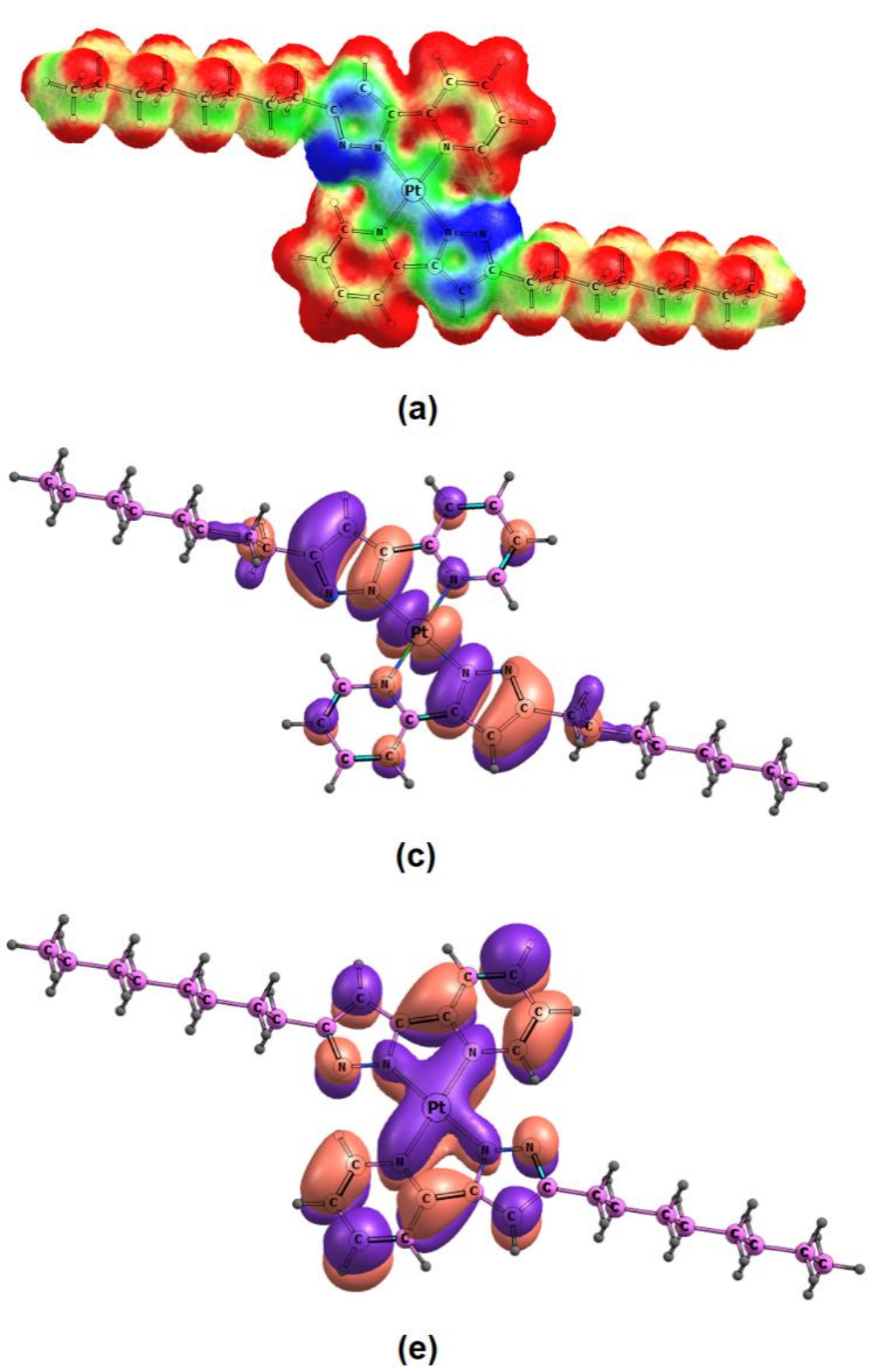

Figure S2. MESP distribution on isodensity surface of value 0.01 a.u. for **(a) Pt(8ppz)$_2$** and **(b) Pt(fppz)$_2$**. Color coding from blue to red is -20.0 to +20.0 kcal/mol. **(c)** HOMO and **(e)** LUMO of **Pt(8ppz)$_2$**; **(d)** HOMO and **(f)** LUMO of **Pt(fppz)$_2$**.

Section S4. Material properties

Thermal Properties

The decomposition temperature quoted at 5% weight loss was found to be 385 and 347 °C for **Pt(8ppz)$_2$** and **Pt(fppz)$_2$**, respectively (**Figure S3a**), where the latter in fact is a sublimation temperature. Under DSC conditions (**Figure S3b**), **Pt(8ppz)$_2$** demonstrated semi-crystalline behaviour with an irreproducible endothermic peak centred at 146 °C, ascribed as a partial melt of a minor polymorph. No corresponding solidification peak was observed in the cooling cycle, indicating the material remained in a single state. Subsequent heating and cooling cycles at 100 °C min^{-1} remained featureless, as did those completed at 50 and 200 °C min^{-1}. To manufacture evaporated neat film devices, the sublimation temperature was estimated using a

gradient vacuum sublimation apparatus (**Figure S4**). At 10^{-6} mbar, a stable sublimation rate was observed for Pt(8ppz)$_2$ and Pt(fppz)$_2$ at 210 and 170 °C, respectively.

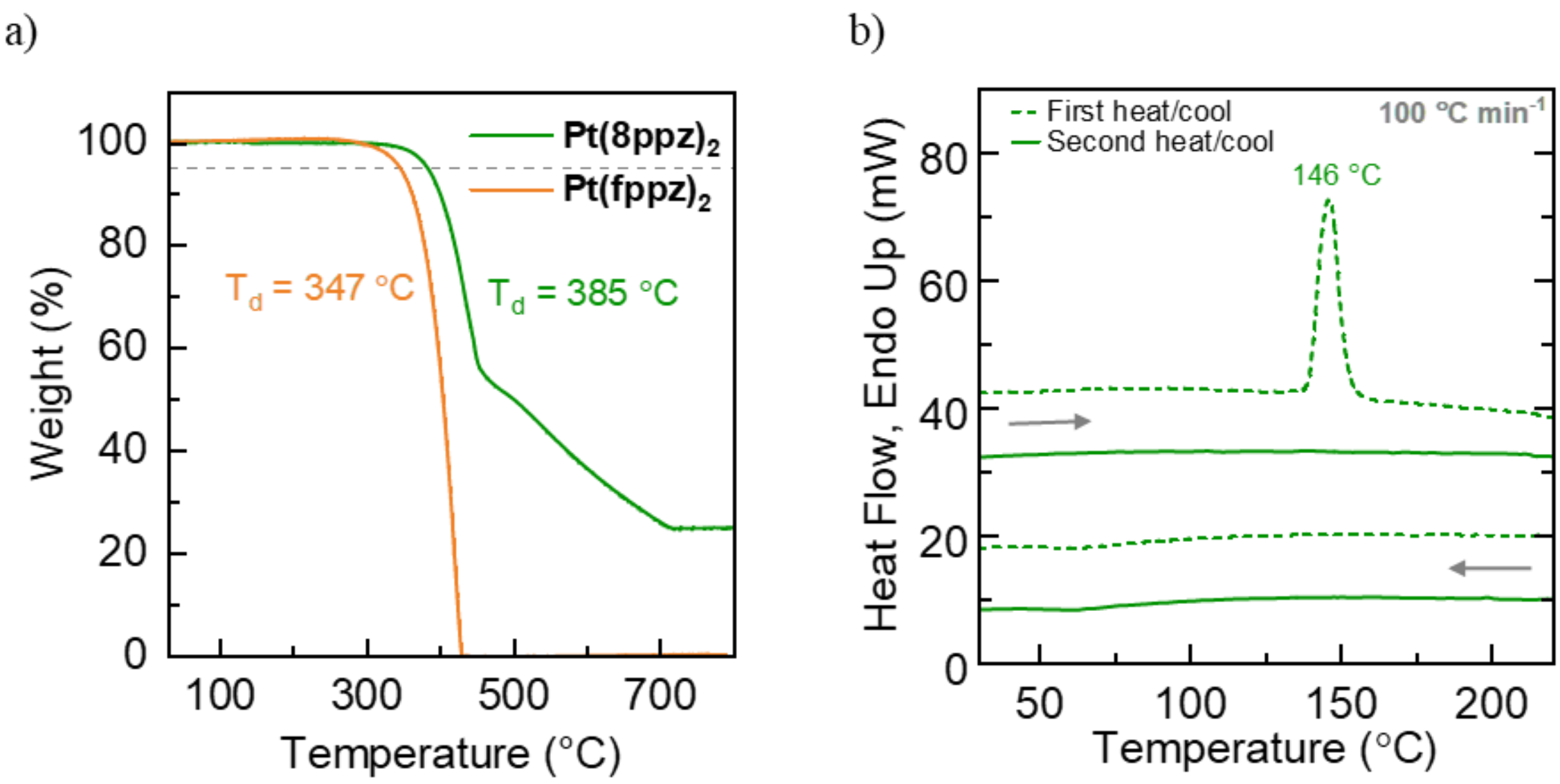

Figure S3. a) TGA traces of Pt(8ppz)$_2$ (green) under $N_{2(g)}$ with a scan rate of 10 °C min^{-1}, showing decomposition temperatures (T_d at 5% weight loss) of 385 °C as opposed to Pt(fppz)$_2$ (brown) with a sublimation temperature at ≈347 °C. **b)** DSC traces of Pt(8ppz)$_2$ at scan rate of 100 °C min^{-1}, showing the first (dashed) and subsequent (solid) heating and cooling cycles with a crystal-crystal transition temperature of 146 °C in the first heating scan, after which the sample remained consistent.

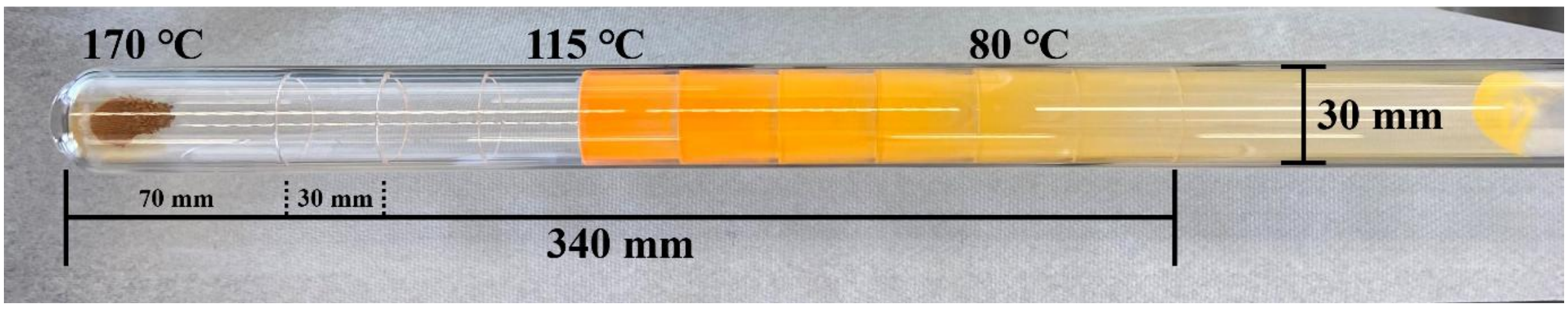

Figure S4. Example purification of Pt(fppz)$_2$ by vacuum sublimation onto segmented quartz, using Creaphys DSU05 system under 4 × 10^{-7} mbar. The outer quartz tube was loaded into an external oven with three independent heating zones used to create a temperature gradient for controlled material collection. Glass wool was used to prevent material drift outside the collection area.[1]

Electrochemical Properties

Pt(fppz)$_2$ was insufficiently soluble to complete electrochemical measurements in solution. While Pt(8ppz)$_2$ also showed low solubility, a combination of cyclic voltammetry (CV) and differential pulse voltammetry (DPV) was able to reveal the first oxidation and reduction potentials (**Figure S3**). The reduction potentials were visualised using CV in THF while the oxidation potential was collected with DPV in DCM. Referencing to ferrocene, the $E_{1/2(red)}$ of Pt(8ppz)$_2$ was –2.35 V, and the oxidation potential 0.68 V, corresponding to an estimated LUMO and HOMO energy of –2.5 and –5.5 eV, respectively, using the work function of –4.8 eV for ferrocene against vacuum level.[2,3]

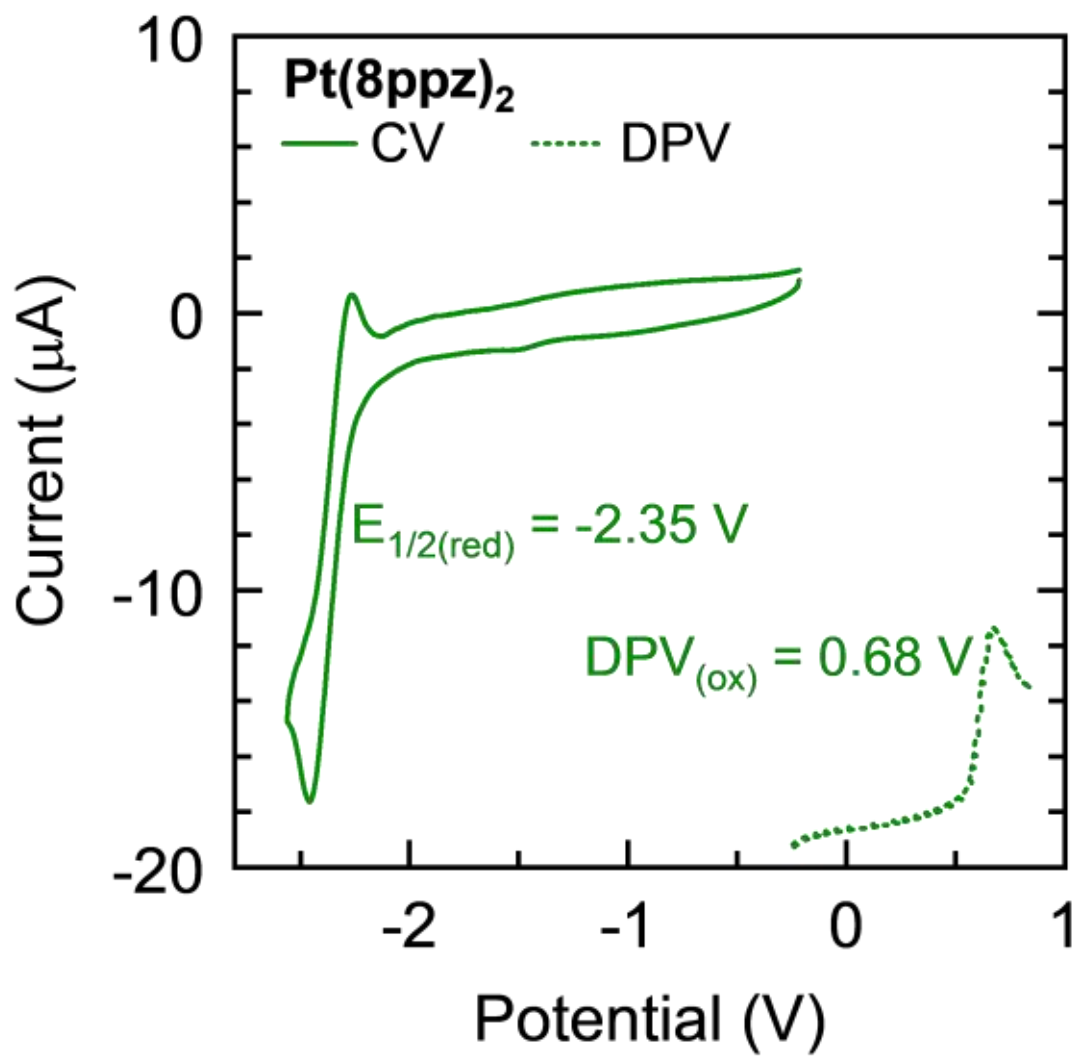

Figure S5. Electrochemical properties of Pt(8ppz)$_2$ in solution, with CV (solid) and DPV (dashed) referenced to ferrocenium/ferrocene couple, revealing the first reduction and oxidation potentials at –2.35 and 0.68 V, respectively.

Section S5. Molecular packing in single crystals and in thin films

Crystal Structure

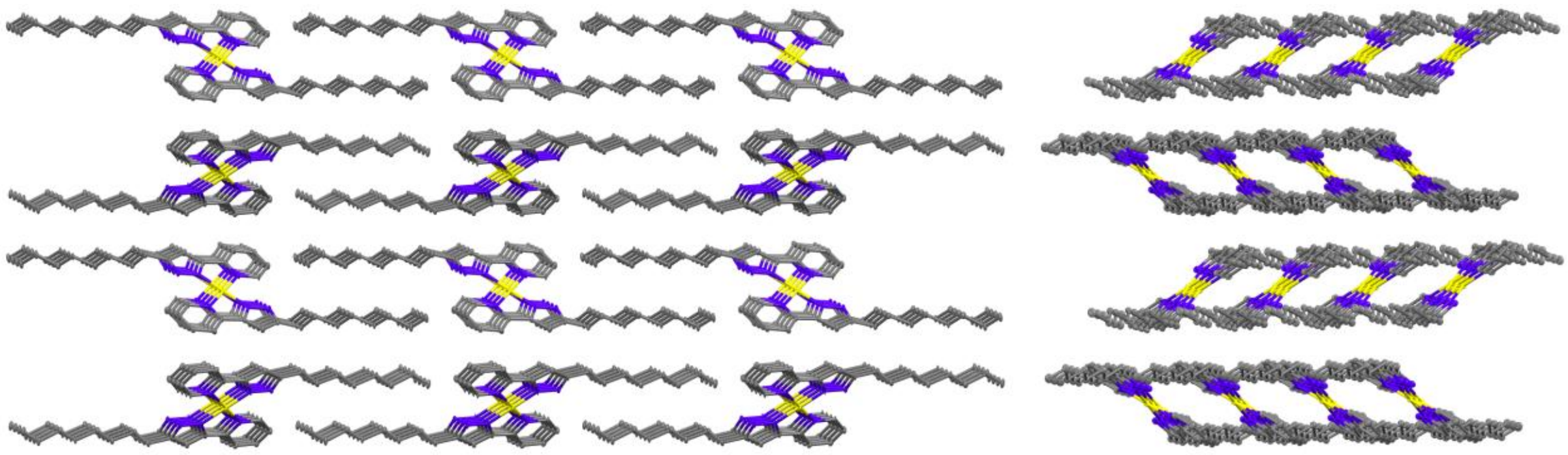

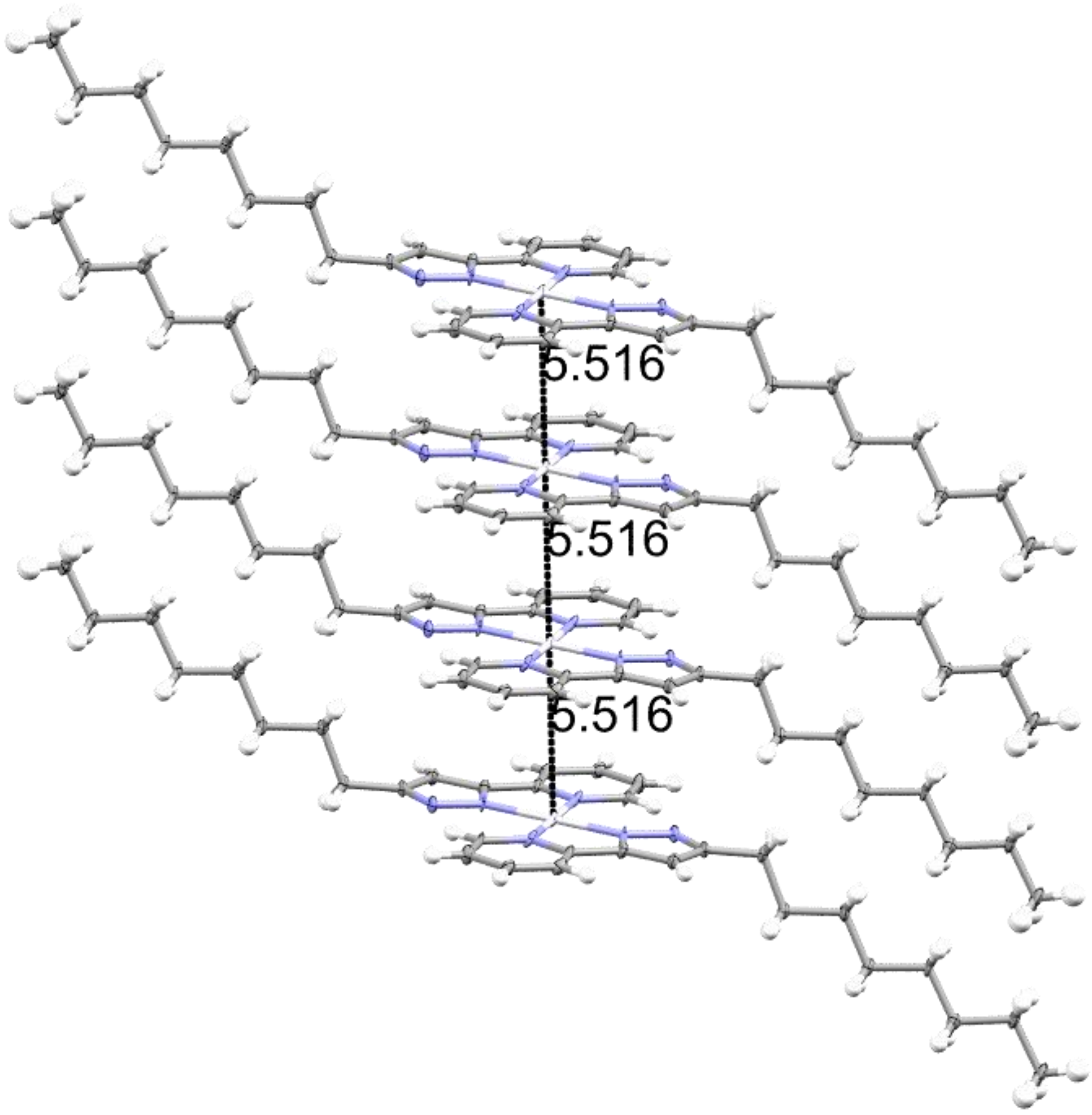

Figure S6. Crystal structure of $Pt(8ppz)_2$, showing Pt-Pt distances of 5.516 Å.

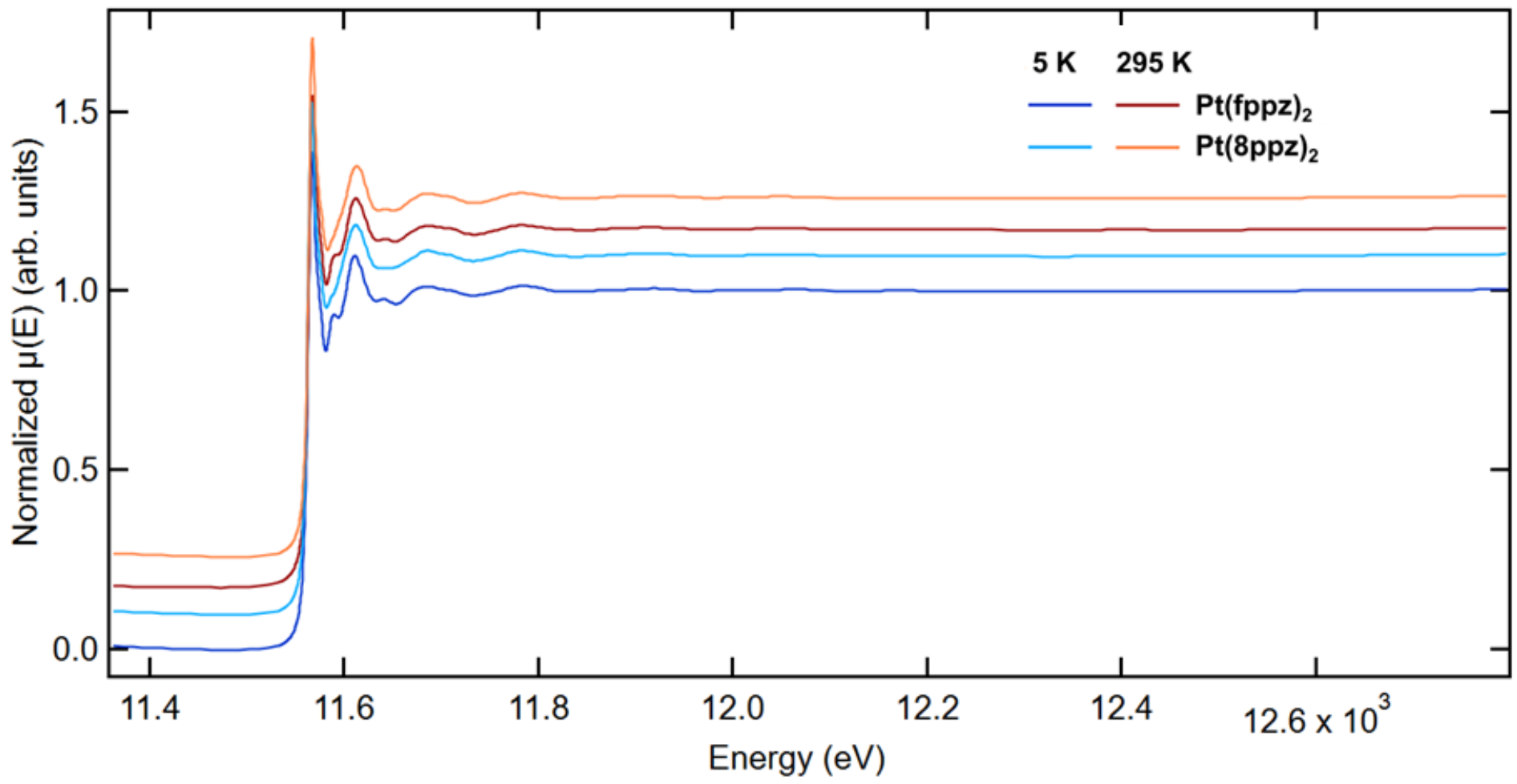

Figure S7. (a) Normalized EXAFS spectra of **Pt(fppz)₂** and **Pt(8ppz)₂** under investigation recorded at 5 and 295 K. Spectra have been offset vertically for clarity.

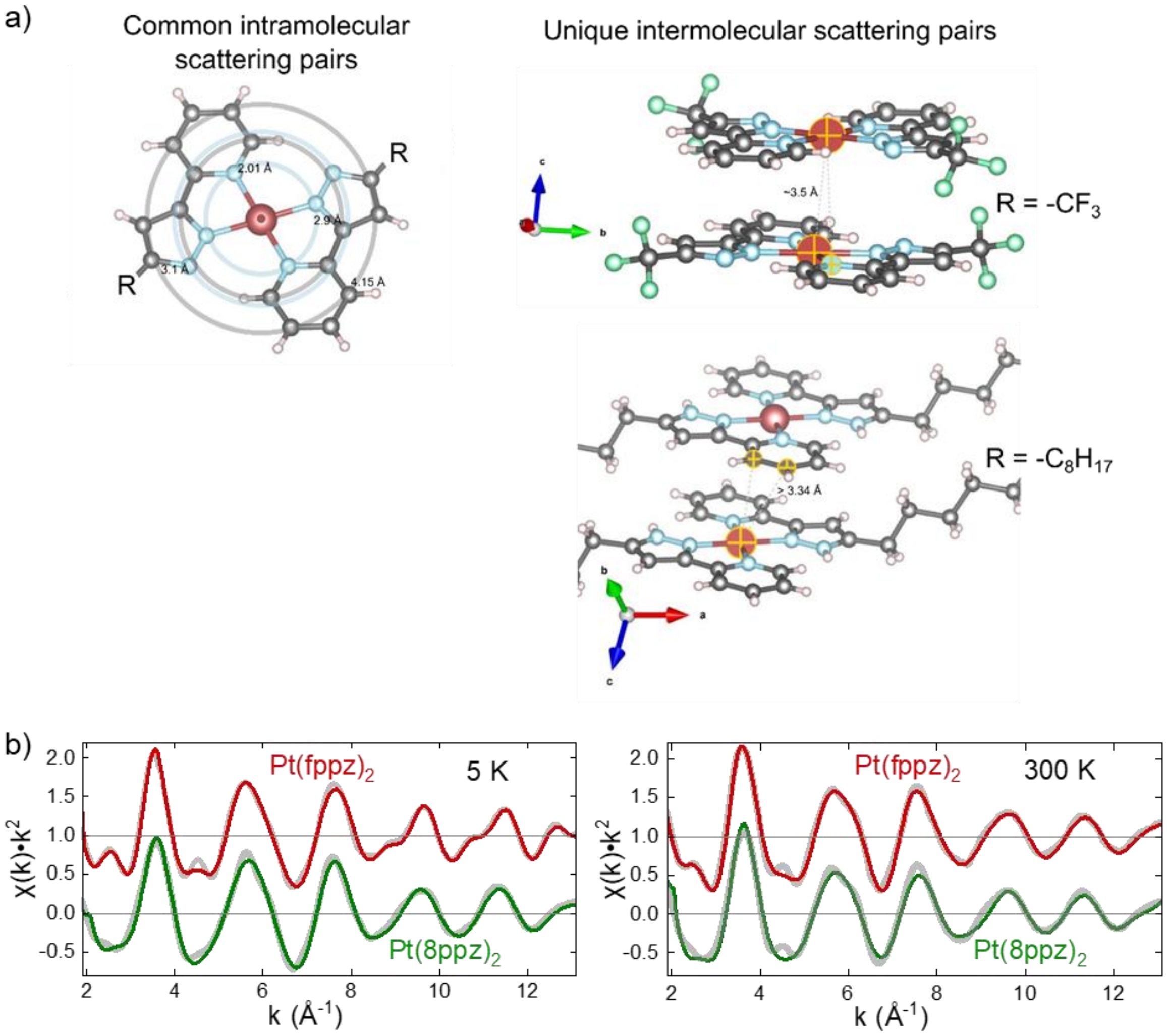

Figure S8. Comparison of common intramolecular fine structure and unique intermolecular fine structure found in the Pt(II) complexes with different R groups used in the EXAFS fits. Only single scattering pairs are shown. **(b)** The experimental k^2-weighted X(k) EXAFS data (grey line) extracted from the Pt-L3-edge (in Figure **S7**), with the fits (coloured lines) corresponding to the different structural models shown in **(a)**.

Table S1. EXAFS fitting parameters extracted from fits made to spectra recorded at 50 and 295 K. The errors provided in the parentheses align with the last significant figures of the variable fit values. Further fitting details can be found in the Methods section. Paths related to unique intermolecular scattering paths are indicated with '*' and the path type is be tracked as: S – single scattering pair; OT – obtuse triangle; FS – forward scattering through sample.

Sample	Path type	N	R (Å)	Σ ($Å^2$)	$ΔE_0$ (eV)	$S0_2$	R-factor
**$Pt(fppz)_2$** (T = 5 K)	Pt-N (S)	4	2.022(6)	0.00169(5)	13.923	0.82(11)	0.005
	Pt-C (S)	4	2.91(8)	0.0027(2)			
	Pt-C (S)	2	3.026(9)	0.0026(5)			
	Pt-N-C (OT)	2	3.095(9)	0.003(1)			
	Pt-N-C (OT)	8	3.159(1)	0.003(1)			
	Pt-N-N (OT)	4	3.205(1)	0.004(1)			
	t-N (S)	4	3.22(1)	0.0017(2)			
	Pt-N (S)	2	3.61(1.2)	0.0031(7)			
	**Pt-Pt (S)***	**1.6(3)**	**3.32(1)**	**0.0038(3)**			
	Pt-N-N (FS)*	3.9(7)	4.15(1.3)	0.008(4)			
**$Pt(8ppz)_2$** (T = 5 K)	Pt-N (S)	4	2.024(2)	0.0023(4)	14.278	0.9	0.004
	Pt-C (S)	4	2.908(4)	0.0027(2)			
	Pt-C (S)	2	3.023(4)	0.0026(5)			
	Pt-N-C (OT)	2	3.093(4)	0.003(1)			
	Pt-N-C (OT)	8	3.158(4)	0.003(1)			
	Pt-N-N (OT)	4	3.203(4)	0.004(1)			
	Pt-N (S)	4	3.218(4)	0.0017(2)			
	Pt-N (S)	2	3.61(5)	0.0031(7)			
	Pt-N (S)*	0.7(2)	4.11(6)	0.0038(3)			
	Pt-C (S)*	1.1(4)	4.23(8)	0.008(4)			

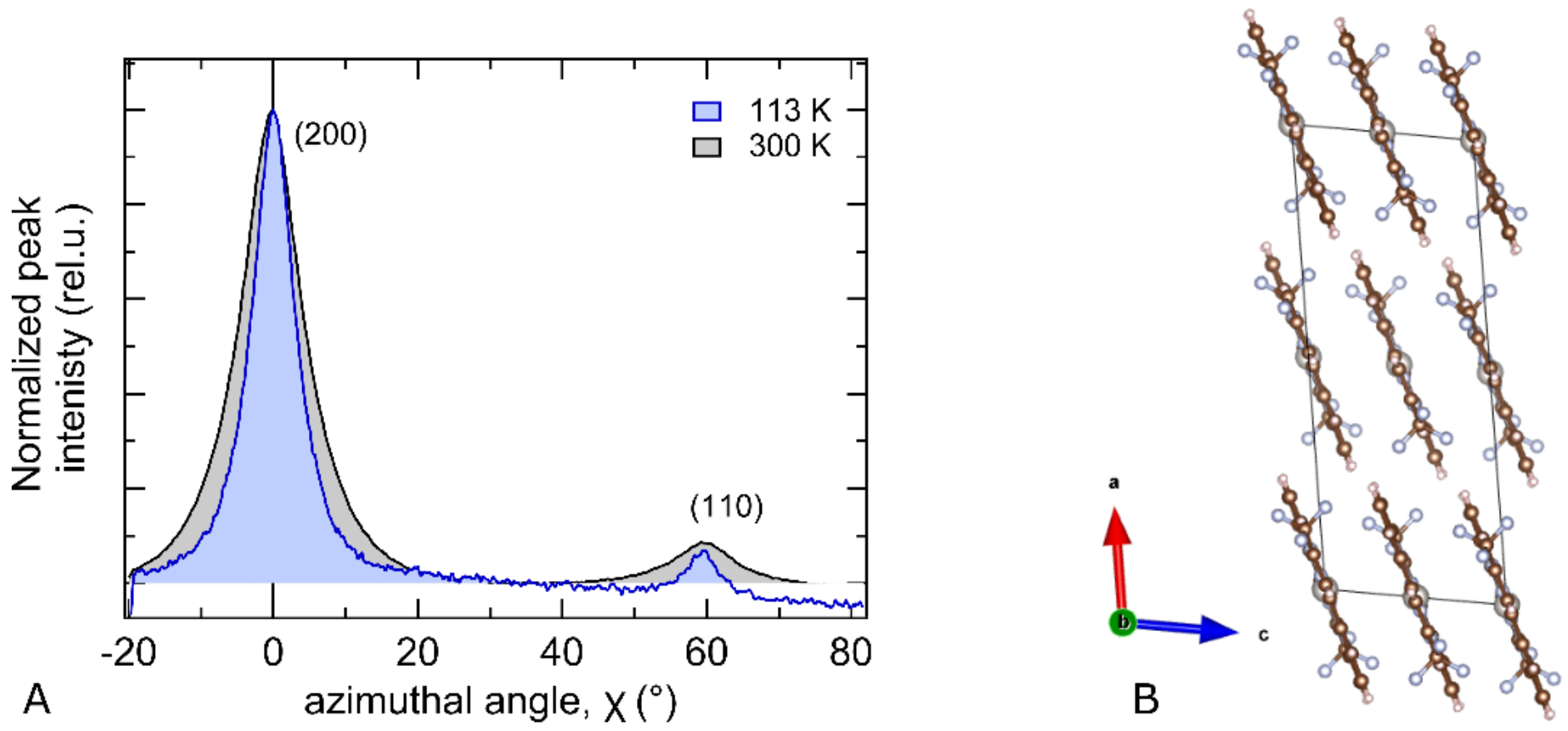

Figure S9. a) The intensity of the (200) scattering planes measured from the Pt(fppz)$_2$ thin film (after background subtraction and without correcting for the intersection of the Ewald sphere) as a function of azimuthal angle (χ) on the detector. These data have been rescaled relative to their out-of-plane signal. **b)** Illustration of the relative angle of the crystallographic a-axis that is pointing out of plane in the 2D GIWAXS image shown in **Figure 1b** of the main text.

Section S6: Photophysical Properties

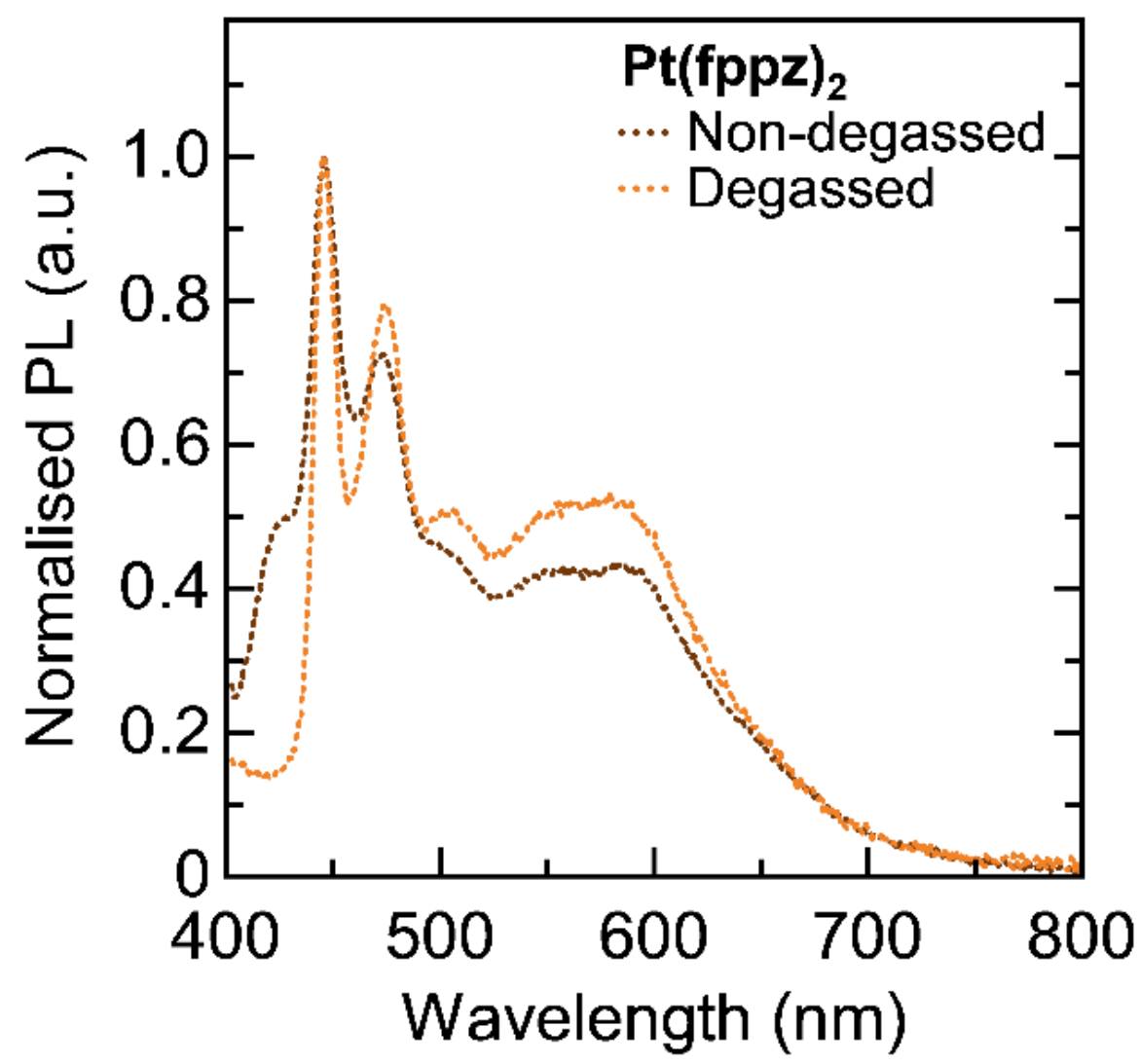

Figure S10. Normalized dilute solution (toluene) PL of Pt(fppz)$_2$ under non-degassed and degassed conditions, showing minimal change due to high degree of aggregates.

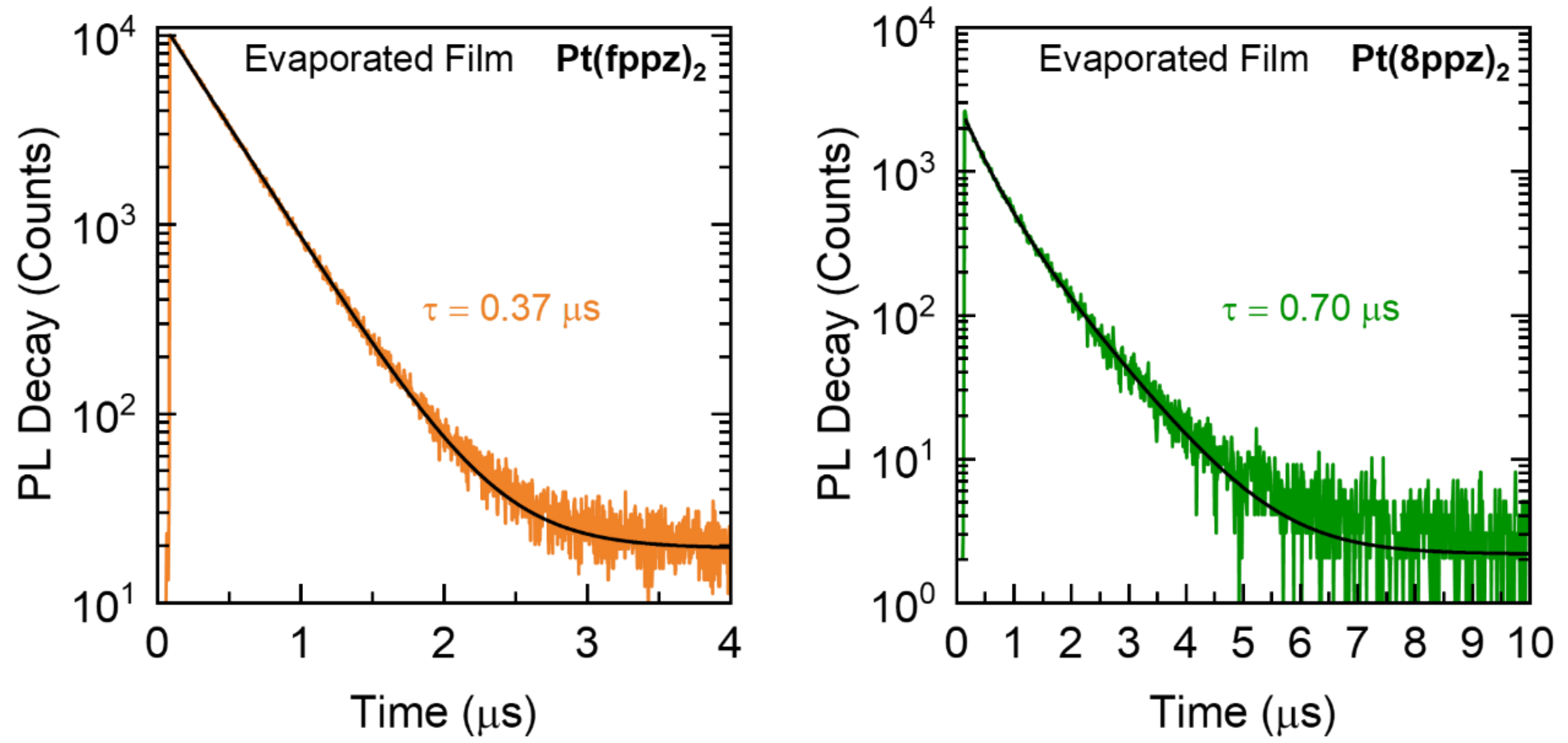

Figure S11. PL decay of evaporated neat films for **Pt(fppz)$_2$** and **Pt(8ppz)$_2$** monitored at 620 nm and 505 nm, respectively.

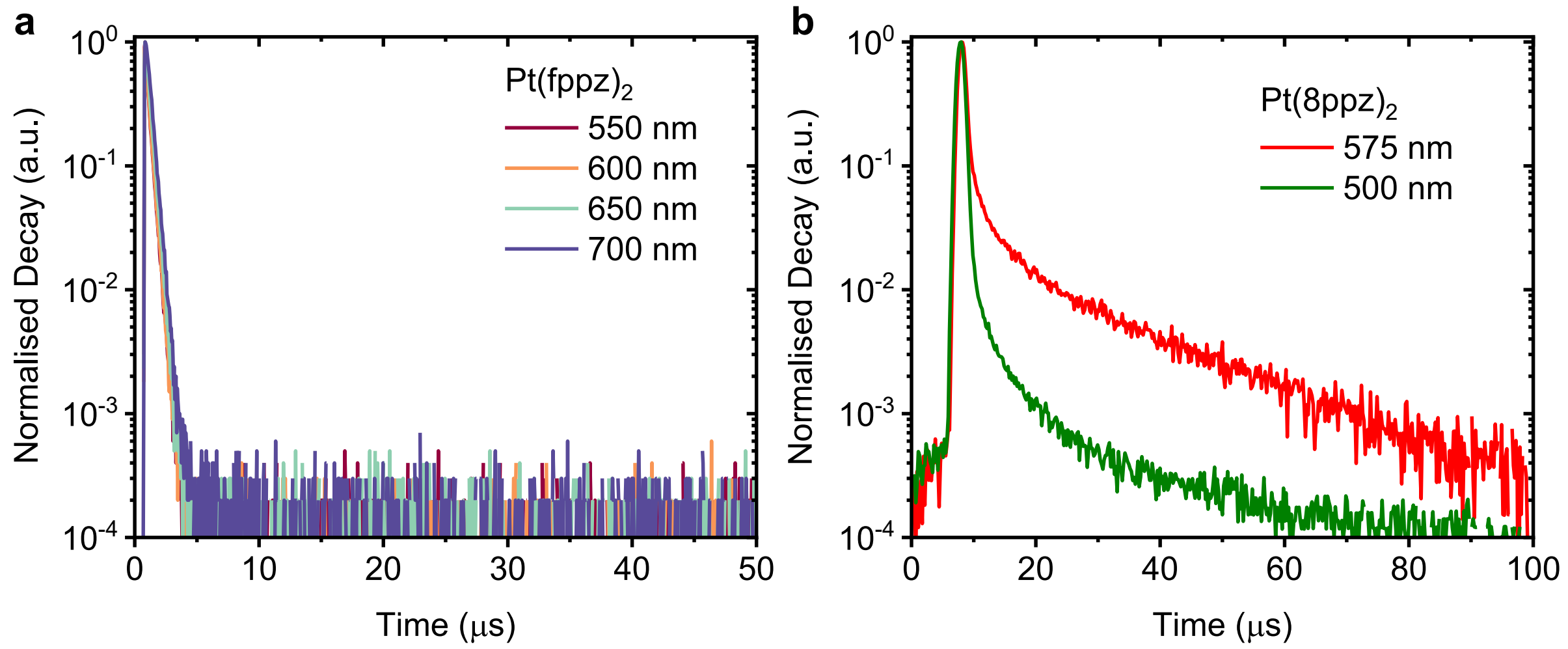

Figure S12. Long-timescale TRPL decay profiles of **a) Pt(fppz)₂** and **b) Pt(8ppz)₂** monitored at selected emission wavelengths. **Pt(fppz)₂** shows wavelength-independent decay with an apparent lifetime of 370 ns, indicating dominant ^{3}MMLCT emission without a significant residual slow component. **Pt(8ppz)₂** exhibits biexponential decay dynamics, consisting of a short-lived component (<1 μs) and a long-lived component of ≈ 8.6 μs, ^{3}MLCT emission and delayed intermolecular ^{3}LC/excimer-like emission, respectively.

Section S7: OLED Characteristics

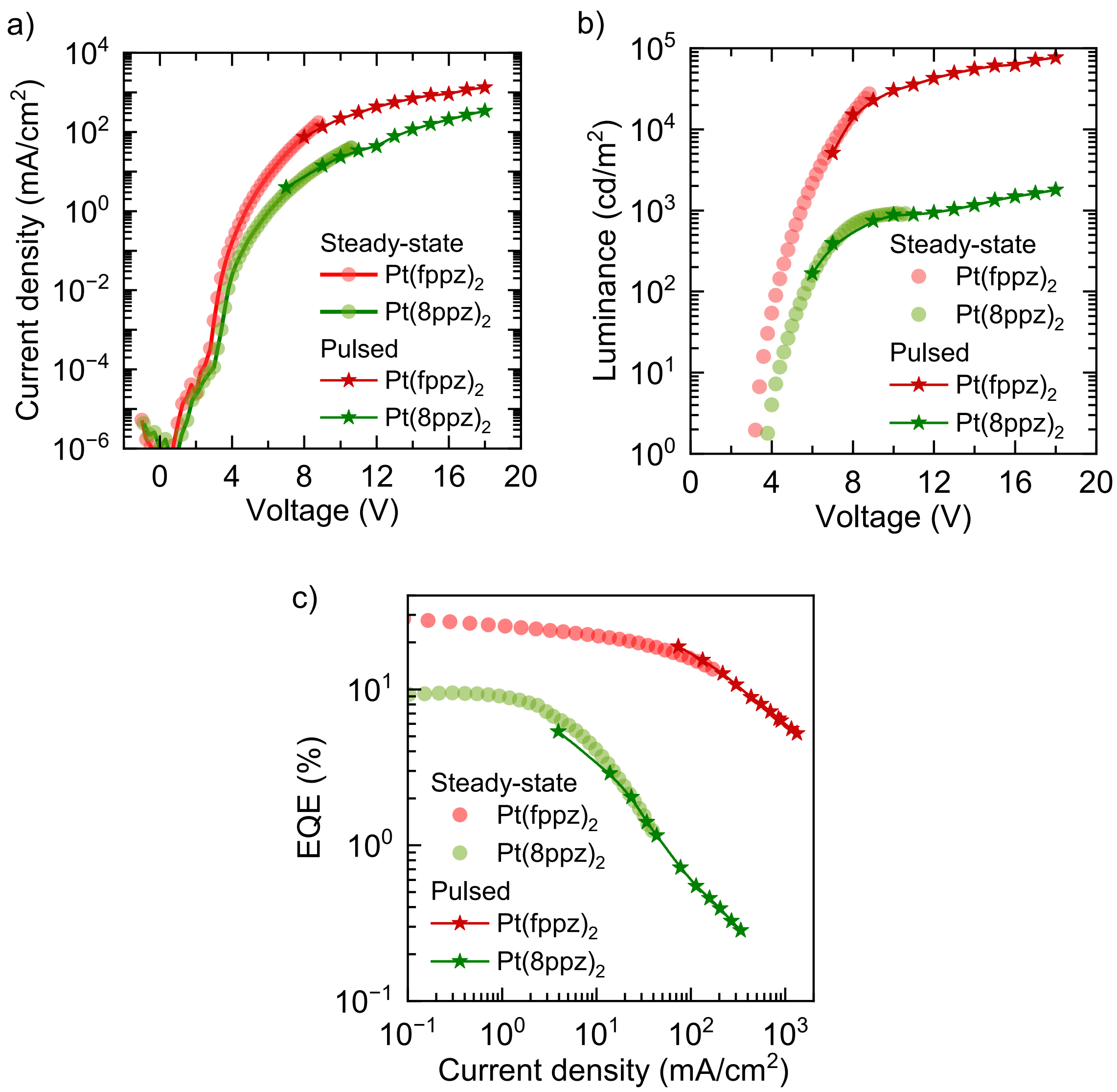

Figure S13. Performance of Pt(fppz)$_2$ and Pt(8ppz)$_2$ based OLEDs under high current densities. Combined steady-state and electrical excitation pulse (under 10 μs voltage pulse) results with comparison of **a)** current density as a function of bias, **b)** luminance vs voltage characteristics and **c)** EQE vs current density of the two OLEDs.

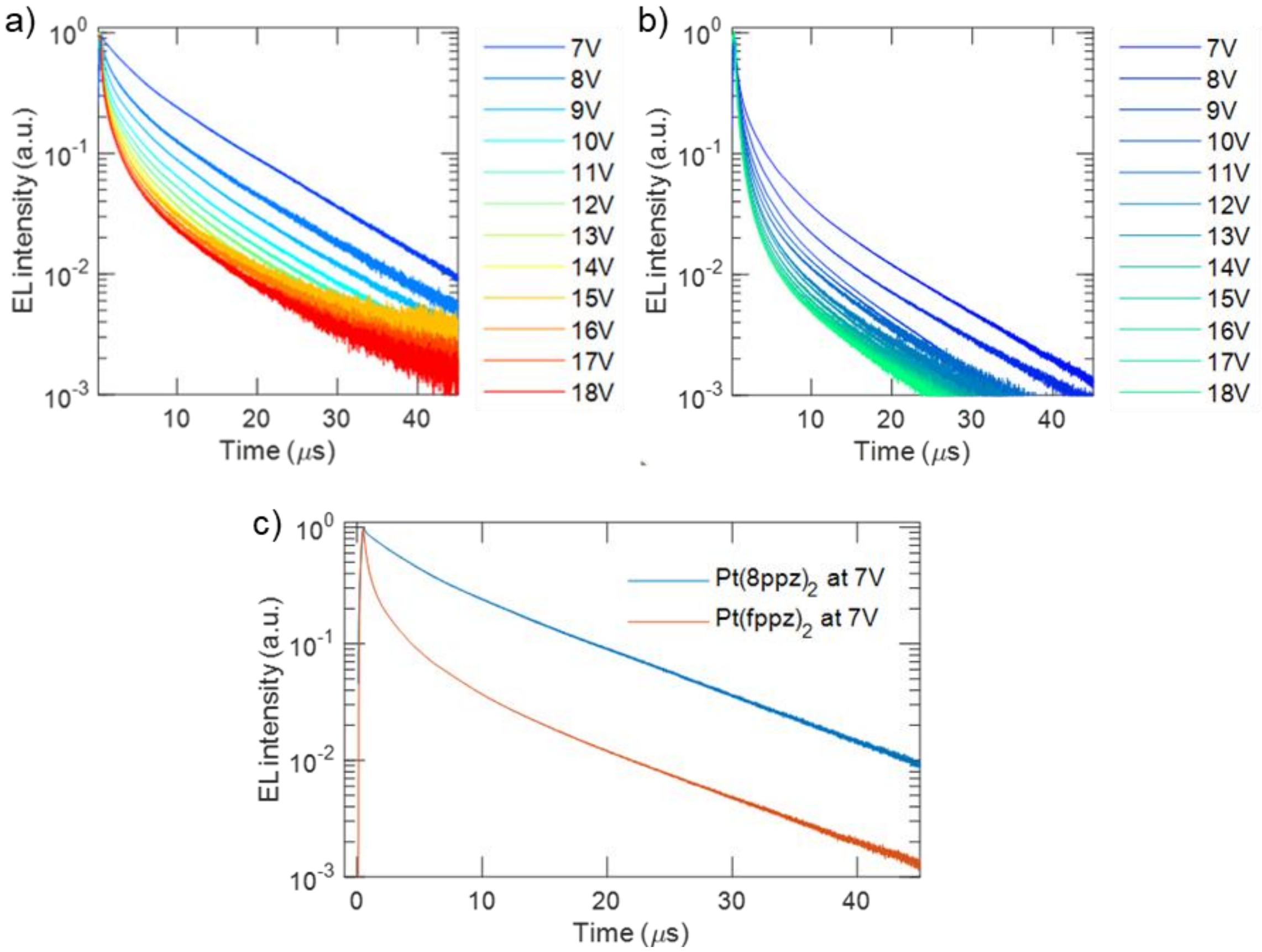

Figure S14. EL decays (pulse-off state) collected with a 500ns excitation pulse. EL decays of **a) $Pt(8ppz)_2$** and **b) $Pt(fppz)_2$** based OLEDs. With increasing voltages, EL decays from both OLEDs get faster due to exciton annihilation processes. **c)** Comparison of EL decays of $Pt(8ppz)_2$ and $Pt(fppz)_2$ based OLEDs at 7 V. The decay from $Pt(fppz)_2$ was short-lived, compared to $Pt(8ppz)_2$ analogous to their PL decays.

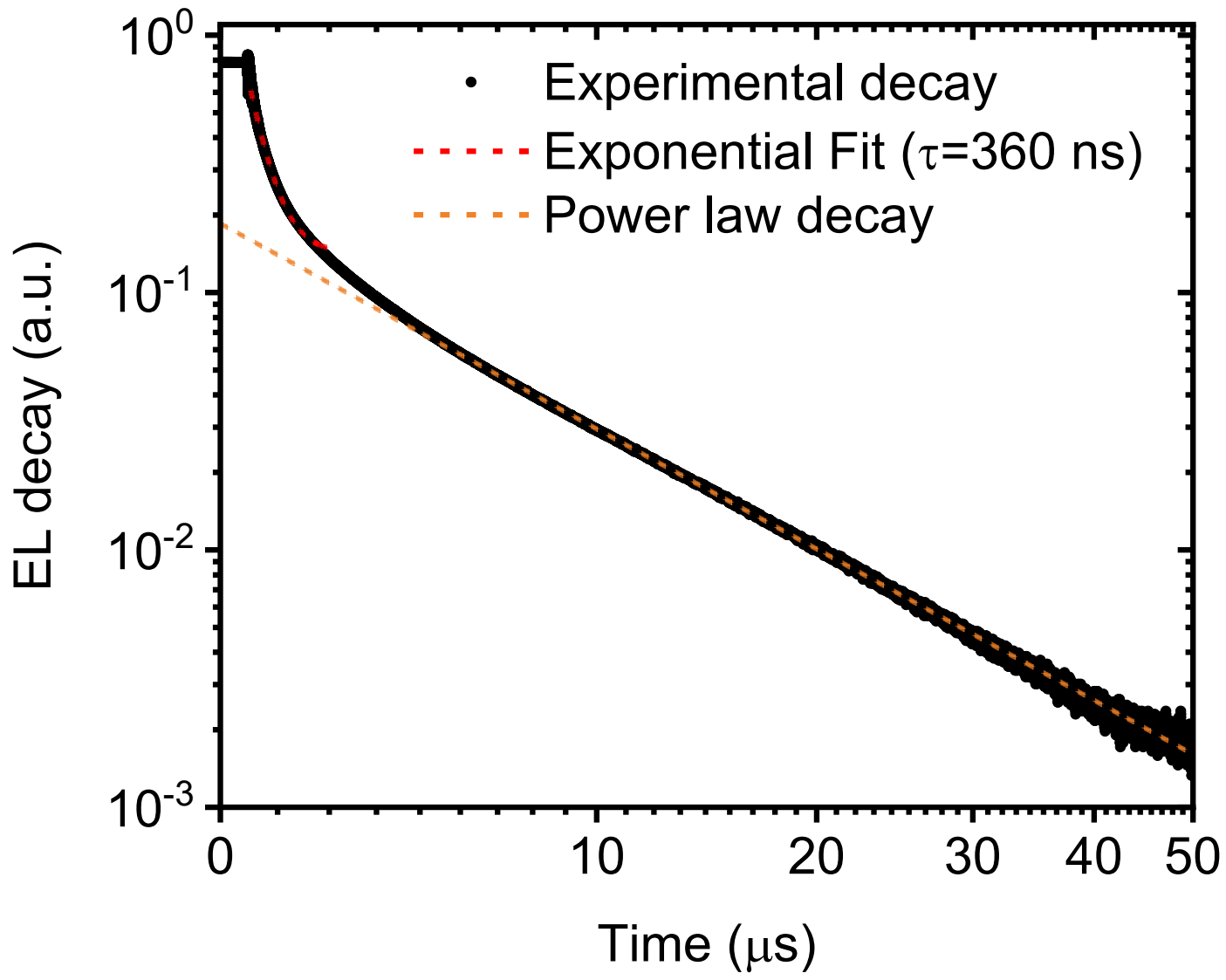

Figure S15. Exponential and power-law analysis of the TREL decay of the **$Pt(fppz)_2$** OLED measured at 7 V. The early-time decay is approximated by an exponential component, corresponding to the prompt EL response after electrical excitation. The delayed tail deviates from single-exponential behaviour and is better described by a power-law dependence, consistent with dispersive delayed recombination in the OLED stack.

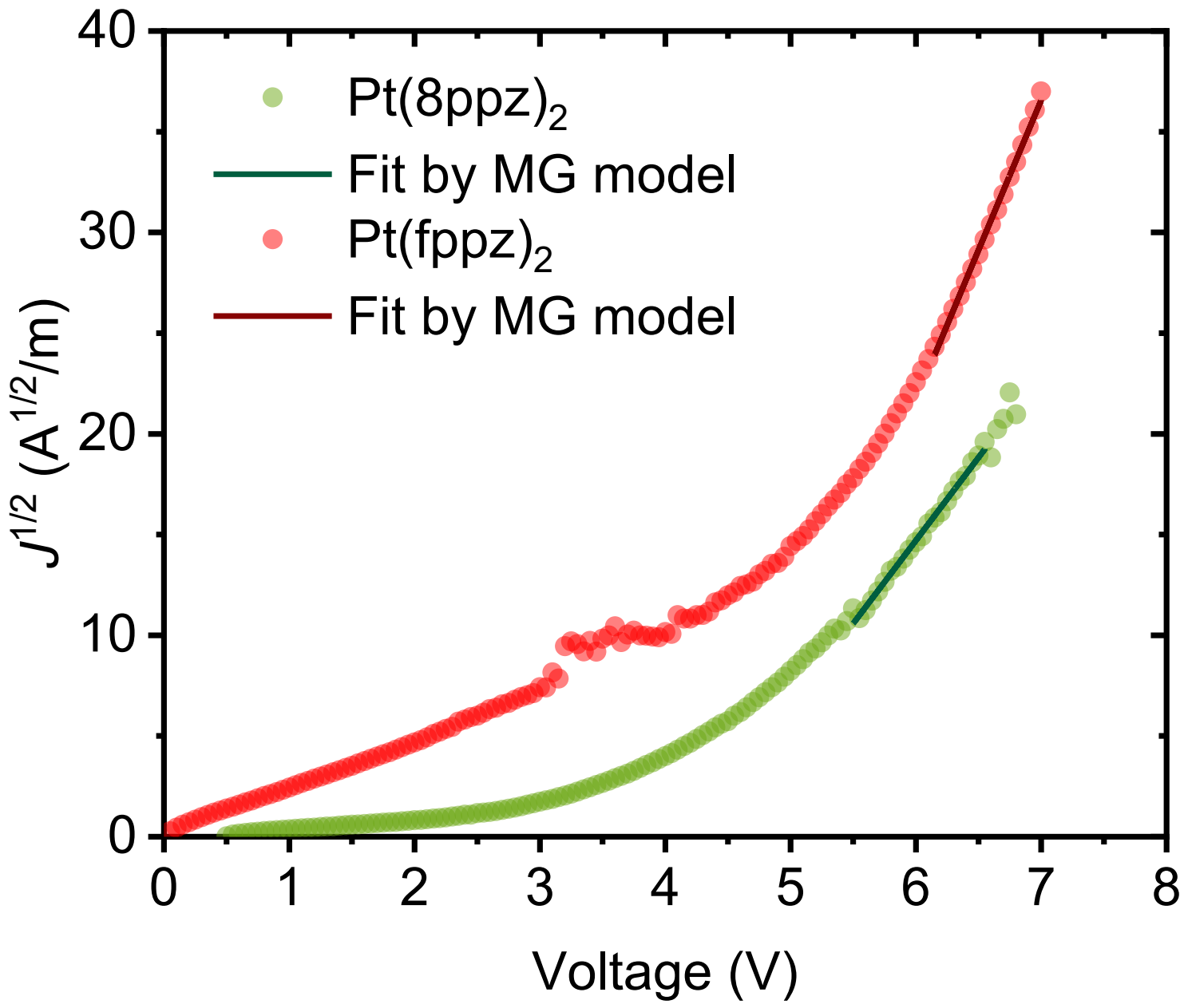

Figure S16. Current density–voltage characteristics of hole-only devices with the structure ITO/MoO_x/Pt complex/MoO_x/Au for **Pt(fppz)₂** and **Pt(8ppz)₂**. Solid lines represent fits using the Mott–Gurney space-charge-limited-current model[4], yielding hole mobilities of 6.5×10^{-5} $cm^2\ V^{-1}\ s^{-1}$ for **Pt(fppz)₂** and 1.9×10^{-5} $cm^2\ V^{-1}\ s^{-1}$ for **Pt(8ppz)₂**.

Section S8: NMR and Mass Spectra

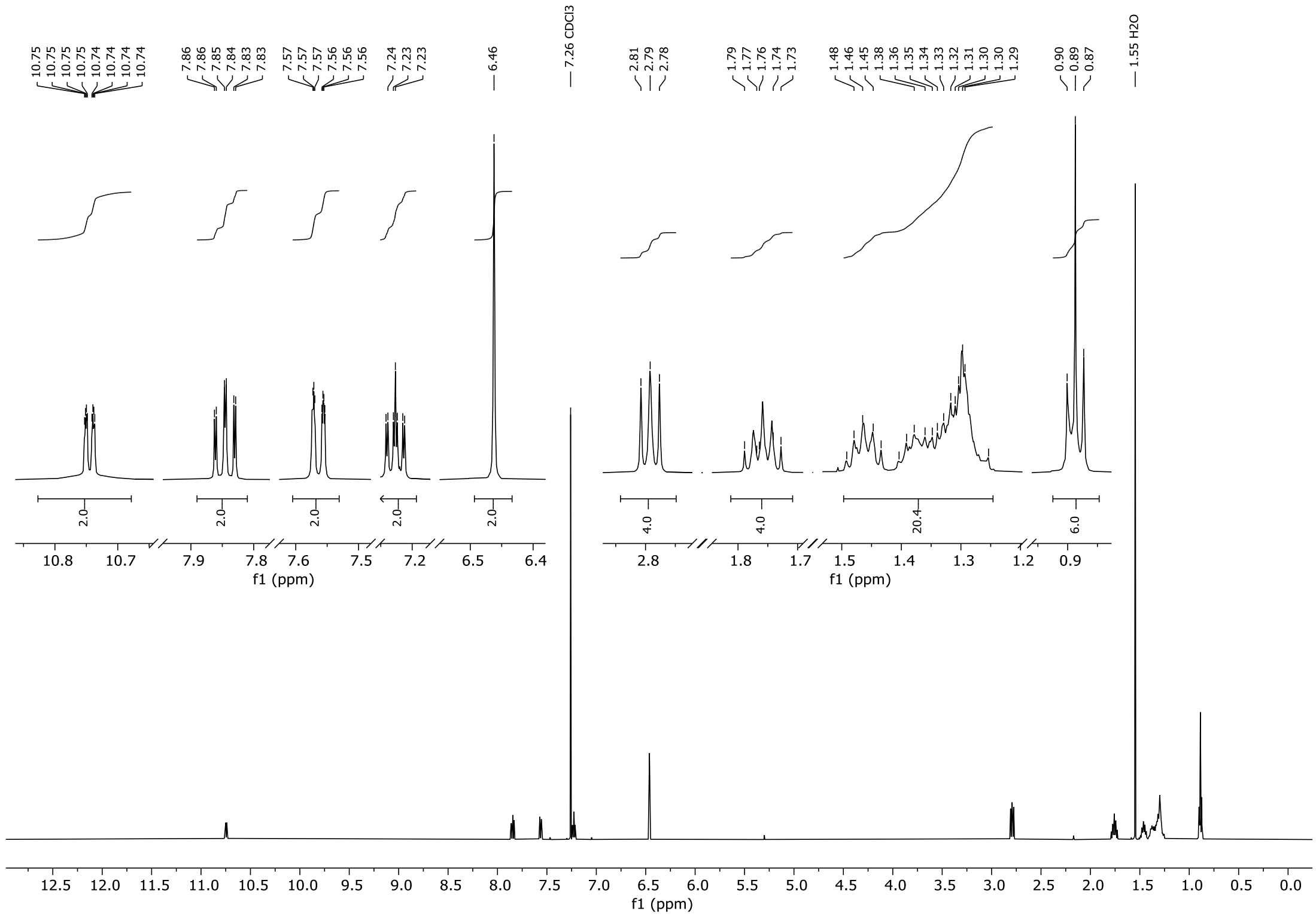

Figure S17. ¹H NMR (500 MHz, $CDCl_3$) of Pt(8ppz)₂.

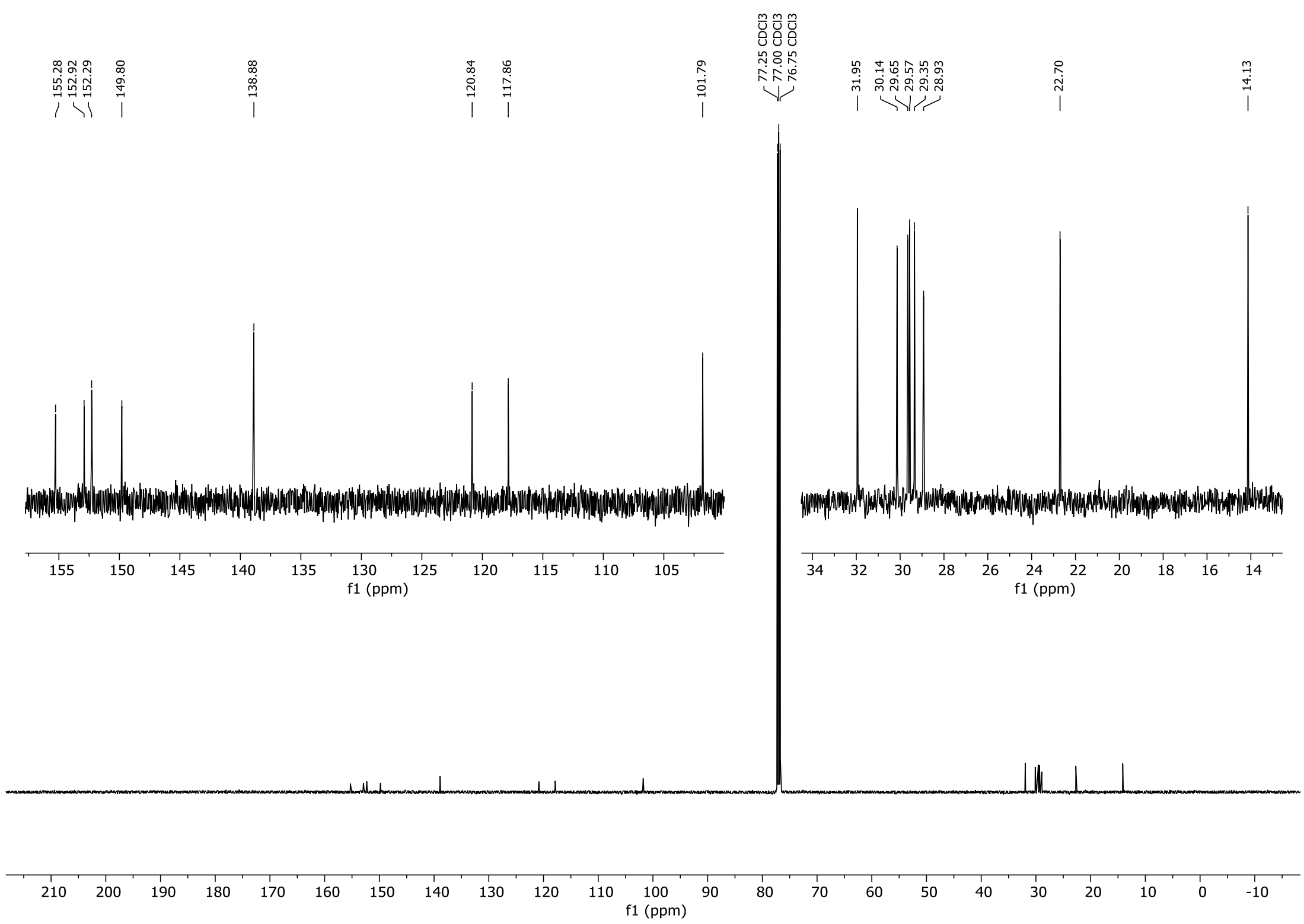

Figure S18. ^{13}C NMR (126 MHz, $CDCl_3$) of $Pt(8ppz)_2$.

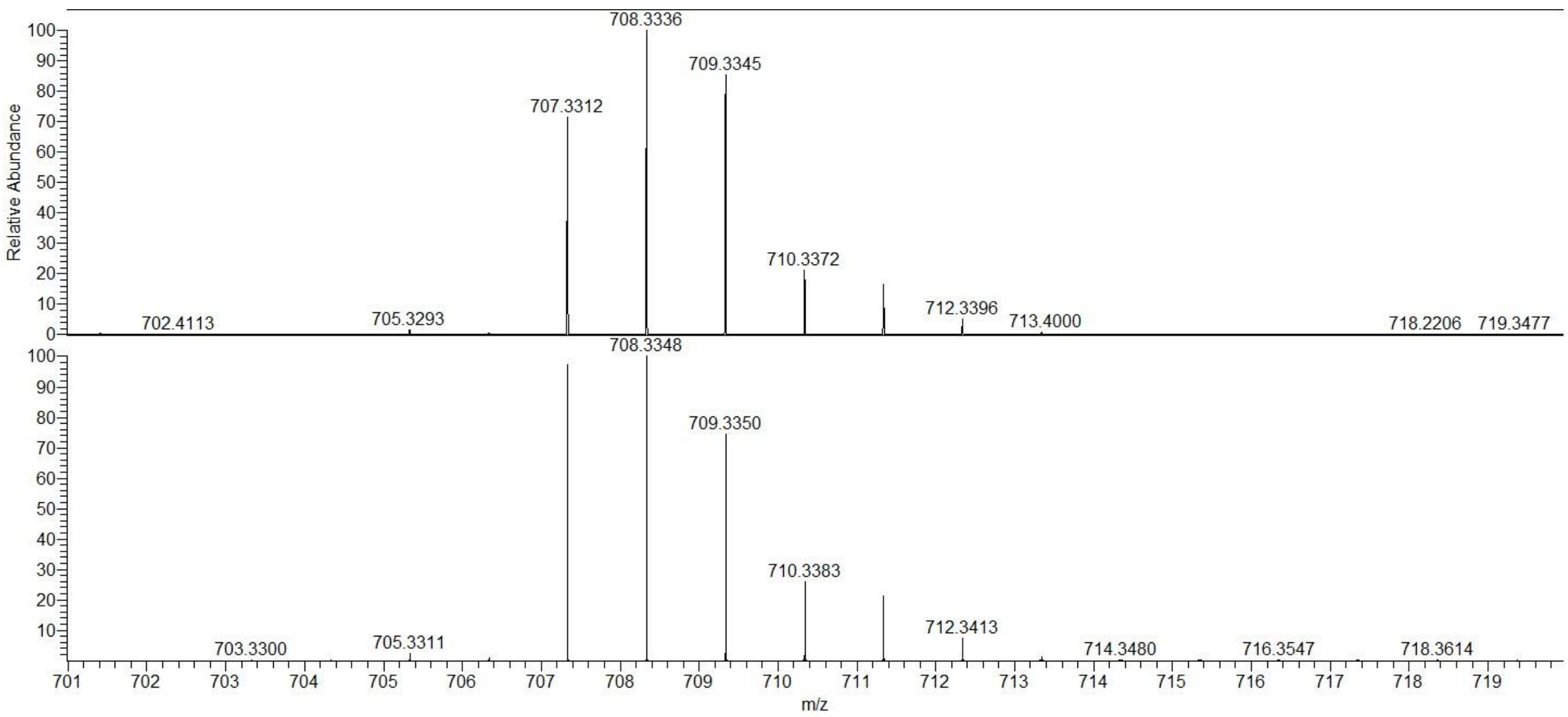

Figure S19. Observed isotope patterns of HRMS of $Pt(8ppz)_2$ (top), matching with theoretical prediction (bottom).

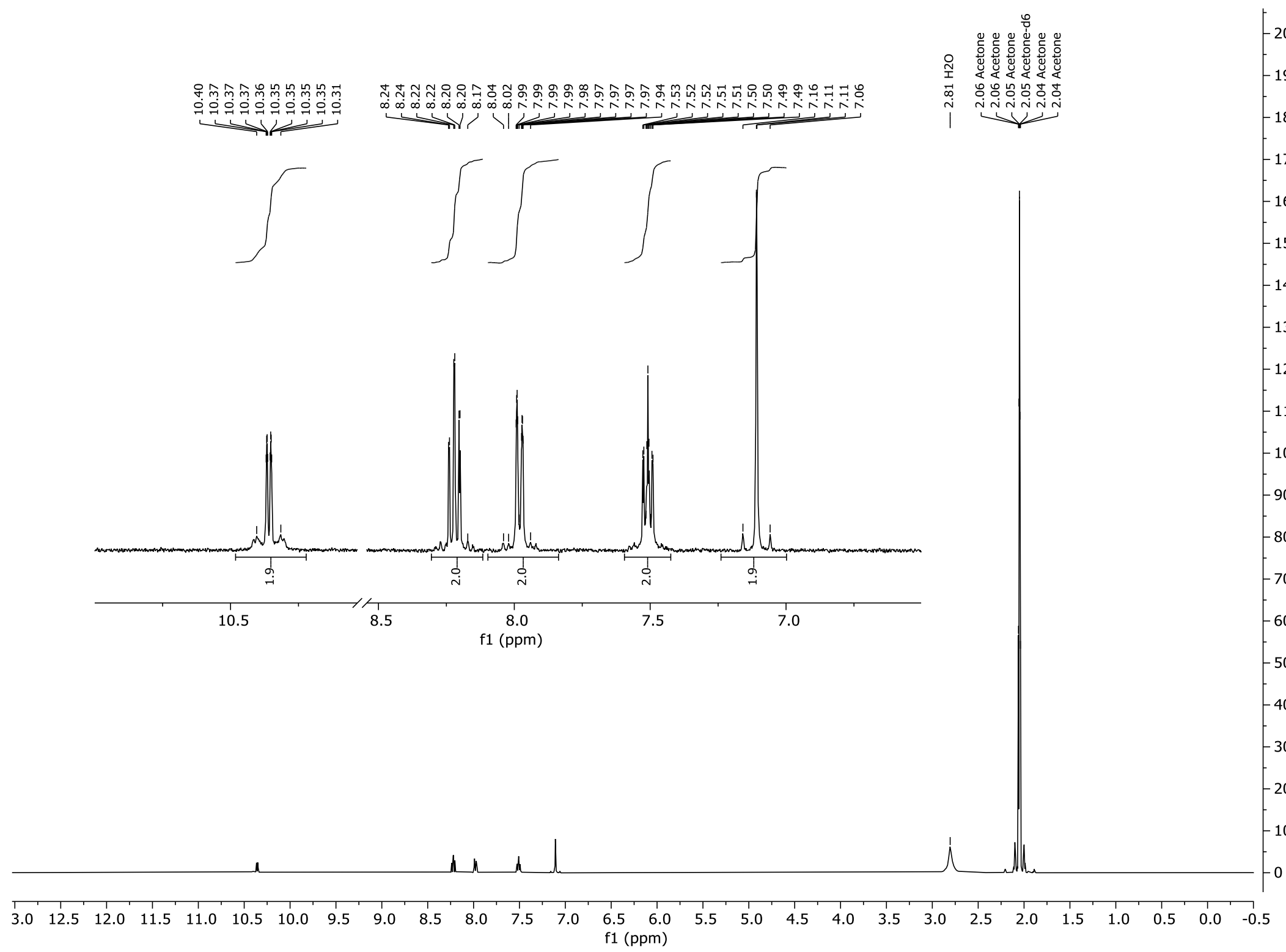

Figure S20. ^{1}H NMR (400 MHz, acetone-d_6) of $Pt(fppz)_2$.

Section S9: Crystal Structure Coordinates

Pt	-0.194059	7.280450	5.512986
C	-0.838317	4.832763	2.114230
H	-0.280836	4.285404	1.609257
N	-1.117916	6.341272	3.980927
N	1.343643	6.261187	4.720770
C	-2.180833	4.955074	1.803849
H	-2.540639	4.495591	1.078836
C	1.069707	5.520037	3.618172
C	5.482180	5.713697	3.009539
H	5.499326	6.669285	3.174487
H	5.057710	5.565147	2.150053
N	2.663182	6.167997	5.020125
C	-2.427613	6.453391	3.657315
H	-2.971136	7.006370	4.170695
C	11.476354	5.083210	0.716137
H	11.898319	5.148181	1.587552
H	11.345808	4.142998	0.519070
C	-0.337287	5.549159	3.199186
C	6.923333	5.174944	2.968743
H	7.340660	5.335958	3.829469
H	6.893609	4.215191	2.831767
C	7.790087	5.793782	1.884890
H	7.864306	6.748016	2.045103
H	7.358297	5.670568	1.025018
C	3.197530	5.352587	4.086225
C	-2.986703	5.770485	2.587795
H	-3.893345	5.858651	2.397261
C	2.236034	4.920128	3.163902
H	2.350746	4.364732	2.426496
C	4.665404	5.024967	4.103315
H	4.769871	4.065214	4.010328

H	5.029559	5.276899	4.966737
C	9.197555	5.185136	1.830863
H	9.617147	5.298741	2.697708
H	9.115277	4.231645	1.668797
C	10.110323	5.780677	0.767408
H	10.241139	6.723248	0.952716
H	9.681821	5.703461	-0.099057
C	12.410264	5.688944	-0.335190
H	11.983951	5.661394	-1.194918
H	13.226429	5.183680	-0.366884
H	12.606076	6.599087	-0.103457
C	0.450198	9.728137	8.911741
H	-0.107282	10.275496	9.416714
N	0.729797	8.219628	7.045044
N	-1.731761	8.299713	6.305202
C	1.792715	9.605826	9.222122
H	2.152521	10.065309	9.947135
C	-1.457826	9.040863	7.407799
C	-5.870298	8.847203	8.016432
H	-5.887444	7.891615	7.851484
H	-5.445828	8.995753	8.875918
N	-3.051300	8.392903	6.005846
C	2.039495	8.107509	7.368657
H	2.583018	7.554530	6.855276
C	-11.864473	9.477690	10.309834
H	-12.286438	9.412719	9.438419
H	-11.733926	10.417902	10.506902
C	-0.050831	9.011741	7.826786
C	-7.311451	9.385956	8.057228
H	-7.728778	9.224942	7.196503
H	-7.281727	10.345709	8.194204
C	-8.178205	8.767118	9.141081
H	-8.252424	7.812884	8.980869
H	-7.746415	8.890332	10.000953

C	-3.585648	9.208313	6.939746
C	2.598585	8.790415	8.438176
H	3.505226	8.702249	8.628710
C	-2.624152	9.640772	7.862069
H	-2.738865	10.196168	8.599475
C	-5.053522	9.535933	6.922656
H	-5.157989	10.495686	7.015644
H	-5.417677	9.284001	6.059234
C	-9.585673	9.375764	9.195109
H	-10.005265	9.262159	8.328263
H	-9.503395	10.329255	9.357174
C	-10.498441	8.780223	10.258564
H	-10.629258	7.837652	10.073256
H	-10.069939	8.857439	11.125028
C	-12.798382	8.871956	11.361161
H	-12.372069	8.899506	12.220889
H	-13.614547	9.377220	11.392855
H	-12.994194	7.961813	11.129428

Section S10: References

1. Morgan, N. T., et al. Mechanism for the separation of organic semiconductors via thermal gradient sublimation, *Org. Electronics* **24**, 212–218 (2015).
2. Lo, S.-C., et al. High-triplet-energy dendrons: enhancing the luminescence of deep blue phosphorescent iridium (III) complexes, *J. Am. Chem. Soc. J. Am. Chem. Soc.* **131**, 16681–16688 (2009).
3. D'Andrade, B. W., et al. Relationship between the ionization and oxidation potentials of molecular organic semiconductors *Org. Electronics* **6**, 11–20 (2005).
4. P N Murgatroyd. Theory of Space-Charge-Limited Current Enhanced by Frenkel Effect. *J Phys. D. Appl. Phys.*, **3**, 151 (1970).
